# The timing of HIV-1 infection of cells that persist on therapy is not strongly influenced by replication competency or cellular tropism of the provirus

**DOI:** 10.1371/journal.ppat.1011974

**Published:** 2024-02-29

**Authors:** Sarah B. Joseph, Melissa-Rose Abrahams, Matthew Moeser, Lynn Tyers, Nancie M. Archin, Olivia D. Council, Amy Sondgeroth, Ean Spielvogel, Ann Emery, Shuntai Zhou, Deelan Doolabh, Sherazaan D. Ismail, Salim Abdool Karim, David M. Margolis, Sergei Kosakovsky Pond, Nigel Garrett, Ronald Swanstrom, Carolyn Williamson

**Affiliations:** 1 Department of Microbiology and Immunology, University of North Carolina at Chapel Hill, Chapel Hill, North Carolina, United States of America; 2 Lineberger Comprehensive Cancer Center, University of North Carolina at Chapel Hill, Chapel Hill, North Carolina, United States of America; 3 UNC HIV Cure Center and Department of Medicine, University of North Carolina at Chapel Hill, Chapel Hill, North Carolina, United States of America; 4 Division of Medical Virology, Institute of Infectious Diseases and Molecular Medicine, Faculty of Health Sciences, University of Cape Town, Cape Town, South Africa; 5 Centre for the AIDS Programme of Research in South Africa (CAPRISA), University of KwaZulu- Natal, Durban, South Africa; 6 Department of Epidemiology, Mailman School of Public Health, Columbia University, New York, New York, United States of America; 7 Institute for Genomics and Evolutionary Medicine, Temple University, Philadelphia, Pennsylvania, United States of America; 8 Discipline of Public Health Medicine, School of Nursing and Public Health, University of KwaZulu-Natal, Durban, South Africa; 9 Department of Biochemistry and Biophysics, University of North Carolina at Chapel Hill, Chapel Hill, North Carolina, United States of America; 10 National Health Laboratory Services of South Africa, Johannesburg, South Africa; National Institute for Communicable Diseases, SOUTH AFRICA

## Abstract

People with HIV-1 (PWH) on antiretroviral therapy (ART) can maintain undetectable virus levels, but a small pool of infected cells persists. This pool is largely comprised of defective proviruses that may produce HIV-1 proteins but are incapable of making infectious virus, with only a fraction (~10%) of these cells harboring intact viral genomes, some of which produce infectious virus following ex vivo stimulation (i.e. inducible intact proviruses). A majority of the inducible proviruses that persist on ART are formed near the time of therapy initiation. Here we compared proviral DNA (assessed here as 3’ half genomes amplified from total cellular DNA) and inducible replication competent viruses in the pool of infected cells that persists during ART to determine if the original infection of these cells occurred at comparable times prior to therapy initiation. Overall, the average percent of proviruses that formed late (i.e. around the time of ART initiation, 60%) did not differ from the average percent of replication competent inducible viruses that formed late (69%), and this was also true for proviral DNA that was hypermutated (57%). Further, there was no evidence that entry into the long-lived infected cell pool was impeded by the ability to use the CXCR4 coreceptor, nor was the formation of long-lived infected cells enhanced during primary infection, when viral loads are exceptionally high. We observed that infection of cells that transitioned to be long-lived was enhanced among people with a lower nadir CD4^+^ T cell count. Together these data suggest that the timing of infection of cells that become long-lived is impacted more by biological processes associated with immunodeficiency before ART than the replication competency and/or cellular tropism of the infecting virus or the intactness of the provirus. Further research is needed to determine the mechanistic link between immunodeficiency and the timing of infected cells transitioning to the long-lived pool, particularly whether this is due to differences in infected cell clearance, turnover rates and/or homeostatic proliferation before and after ART.

## Author summary

The long-lived HIV-1 reservoir remains an obstacle to eradicating HIV-1 infections. Only a small proportion of infected cells that persist during ART can produce infectious virus upon treatment interruption, while most cells contain defective proviruses that may be able to produce proteins but cannot generate infectious virus. We report that a majority of the inducible virus and the viral DNA (vDNA) in these long-lived cells were from cells infected around the time of therapy initiation. There is a significant negative correlation between the fraction of the vDNA in long-lived cells that forms around the time of ART initiation and the nadir of CD4+ T cells, suggesting decreased immune surveillance and/or homeostatic proliferation may play a role in the persistence of infected cells. Knowing a precise time when virus infects cells that will become the long-lived pool will provide opportunities to study the cellular mechanisms that allow HIV-infected cells to persist during long-term ART and to develop approaches for blocking or clearing these cells.

## Introduction

A long-lived viral reservoir is established during untreated HIV-1 infection [1–3] with a small portion of infected cells persisting in individuals on long term antiretroviral therapy (ART). The long-lived HIV-1 reservoir is comprised of intact proviruses capable of reinitiating infection if therapy is stopped. In contrast, the pool of all long-lived infected cells includes inducible replication competent proviruses, defective proviruses and possibly intact proviruses that cannot be induced. When provided with strong ex vivo stimulation, about 1% of proviruses in the pool of long-lived infected cells produce infectious virus [4]. These variants are known as the ‘inducible replication competent reservoir’. Of the remaining noninduced proviruses, approximately 12% are intact and 88% are defective [5]. At present, it is not possible to predict whether an intact provirus can generate rebound virus in vivo after therapy discontinuation. In contrast, defective proviruses are unable to generate infectious virus or viral rebound, but they can be transcriptionally active [6,7] and may contribute to immune activation and associated disease. Thus, the pool of long-lived infected cells contains variants that differ in proviral intactness, sensitivity to ex vivo induction, and the ability to be the source of viral rebound.

While ART can fully suppress viral replication [8], discontinuation of therapy results in the rapid reappearance of virus [9–13]. The rebound virus is largely oligoclonal [14–16] indicating that cells are continuously reactivating and producing virus, and that a small number of cells gives rise to the initial rebound virus. A small fraction of people experience post-treatment control of virus, presumably through immunologic mechanisms [17], and the rare examples of HIV-1 "cure" have been in the context of hemopoietic stem cell transplants using cells that are homozygous for a deletion in the viral coreceptor CCR5 [18,19]. The predominant model for creation of the long-lived viral reservoir is that CD4+ T cells are infected around the time they are transitioning from the activated pool to the more quiescent memory pool [20]. However, infected cells in the long-lived reservoir have often undergone T cell clonal expansion, as shown by the detection of cells with HIV-1 DNA integrated at identical positions [21–23], and the transitioning of these cells through multiple differentiation states [14,24–26].

We have previously shown that replication competent variants do not enter the long-lived reservoir at a uniform rate over the course of infection; instead viral variants replicating around the time of ART initiation make up a disproportionately high fraction of inducible replication competent variants in the reservoir [27]. Similarly, studies amplifying defective proviruses from the pool of long-lived infected cells observed that they are also disproportionately comprised of viruses that were replicating late in untreated infection [28–30], although the percent of long-lived infected cells that were infected near the time of ART initiation varies greatly across people on therapy [27, 30]. However, no study has directly compared the seeding of defective and inducible, replication competent variants into the pool of long-lived infected cells in the same individuals.

In this report we use PBMCs collected during ART to examine when both inducible replication competent proviruses (cultured during ex vivo outgrowth assays) and largely defective proviruses (amplified from PBMC DNA) are seeded into long-lived cells in 18 women from the CAPRISA 002 longitudinal cohort. Participants were untreated for 3–5 years and then initiated ART in accordance with in-country guidelines at the time. We assessed the composition of proviruses present in long-lived cells after 4–5 years on suppressive ART. We find that irrespective of whether we examine inducible outgrowth virus (OGV) or proviral DNA (with or without hypermutations), all forms of proviruses in long-lived cells were established at similar times. On average, 60% of the viral DNA in cells isolated from the blood after approximately 5 years of ART came from viruses that were replicating within the final year prior to therapy initiation (i.e. late). The similar timing of introduction of both replication competent virus and defective proviruses into the pool of long-lived cells indicates that it is a feature of the cell, not the virus, that has the greatest impact on transition of the infected cell into the long-lived pool. Finally, we found that lower nadir CD4+ T cell counts are associated with a higher fraction of cells in this pool being infected near the time of ART initiation, suggesting that loss of CD4+ T cells before therapy, failure to clear infected cells and/or increased proliferation of infected cells contribute to the disproportionate entry of late viruses into the long-lived pool of infected cells.

## Results

### Cohort and sample collection

We previously described the timing of entry into the long-lived reservoir of inducible proviruses (outgrowth virus/OGV) for nine women in the CAPRISA 002 cohort [27]. In the current study we extend this work to 18 women from the CAPRISA 002 cohort and perform the first study examining when defective proviruses enter the long-lived pool relative to OGVs in the same participants.

**Fig 1:**
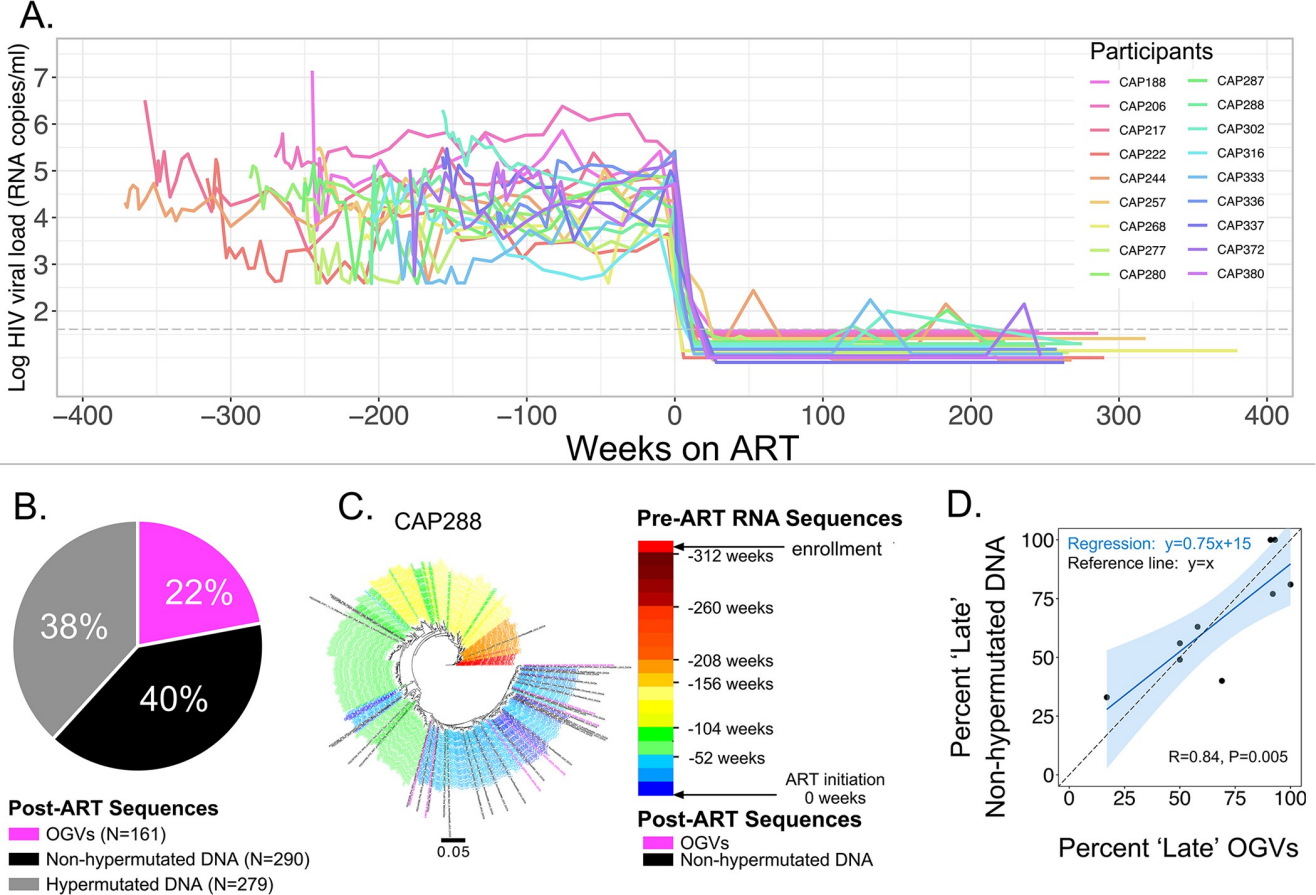
Inducible Proviruses (Outgrowth Viruses, OGV) and Proviral DNA Enter the Pool of Long-Lived Cells at Similar Times. **A**. Longitudinal viral loads of the 18 women from the CAPRISA 002 Cohort whose viral sequences were analyzed. The time of ART initiation in each person is assigned as Week 0 with time increasing to the right (positive values) as time on therapy; the last time point indicated at the right is the point at which blood was donated for reservoir analysis. Conversely, the time prior to ART initiation extends to the left as negative numbers with the leftward-most point representing the sample taken at the time of diagnosis in early infection. Each participant is shown with a different colored line as indicated in the legend. **B**. Identical post-ART sequences were collapsed, and the pie chart represents the percentage of each type of unique viral sequence generated. The source of each sequence and the actual number of those sequences are as indicated along with the color code. **C**. On the left is a phylogenetic tree of partial *env* sequences analyzed for CAP288. On the right is the color code used to annotate the branches in the tree. Sequences generated from pre-ART (viremic) plasma samples within the first year of diagnosis are shown in shades of red, within the last year before therapy initiation are shown in shades of blue, with times between the first and last year shown as orange, yellow, and green. OGV sequences are shown in magenta and non-hypermutated proviral DNA sequences are shown in black. The phylogenetic tree shows the clustering of most of the non-hypermutated proviral DNA (amplified from PBMC DNA) and the OGV sequences with viral sequences replicating in the year before the initiation of therapy. **D.** The fraction of unique post-ART viral sequences that were replicating in the last year before the initiation of therapy is defined as the fraction of long-lived cells infected "late" and represented as a percentage of the total unique sequences sampled. The panel shows the percentage of late sequences in the OGV sample compared to the non-hypermutated proviral DNA sample where each point is one of the nine participants with six or more sequences of both forms. There is a significant correlation between the timing of entry into the pool of long-lived cells containing the two types of viral sequences (non-hypermutated proviral DNA and OGVs; Pearson; R = 0.84, P = 0.005) and the 95% confidence interval around the linear regression that best fits the data includes the line that would be expected if both forms of the reservoir had equal percentages of late reservoir sequences (y = x).

Longitudinal blood plasma samples were collected approximately every six months starting in acute infection and continuing until ART started, with the timing of therapy initiation determined by in-country guidelines at that time (Table 1). The average nadir CD4+ T cell count, excluding acute infection, was 236 cells/μl (IQR 204–278). Deep sequencing of viral RNA from pre-therapy plasma samples (Fig 1A) was performed using a multiplexed cDNA synthesis with a unique molecular identifier in the cDNA primer followed by PCR amplification [31]. We concentrated on four amplicons (‘NEF1’, ‘ENV2’, ‘ENV3’ and ‘ENV4’) in the 3’ half of the genome that gave the strongest and most reproducible signal of evolutionary divergence over time (S1 Fig). This allowed us to use longitudinal pre-therapy sequences to generate an evolutionary timeline to estimate when cells in the long-lived infected pool were infected prior to the initiation of therapy. To confirm that sequences located in the 3’ half of the genome were sufficient for phylogenetic dating, we compared estimates of OGV age (S2A Fig) or proviral DNA age (S2B Fig) when generated with or without a *gag* amplicon in the 5’ half genome. We observed that estimates of when OGVs integrated were similar when generated from sequences with and without the inclusion of *gag*. While proviral DNA sequences displayed a similar pattern, we cannot make robust conclusions about them given the relatively small number of full proviral genomes analyzed (N = 9) from a few participants (N = 4).

**Table 1:** Clinical information for the eighteen women from the CAPRISA 002 cohort.

Patient	Weeks untreated	Weeks on ART	CD4 nadir	Log VL before therapy	Percent long-lived infected cells infected in the year before ART initiation (N = Unique sequence†)					Percent long-lived infected cells infected in the year after transmission	Percent long-lived infected cells in detectable cell clones	sCD4 sens. of OGVs (N = Unique OGVs)	Coreceptor usage of viruses in long-lived infected cells	ART regimen
					OGVs	Total proviral DNA	Hypermut. proviral DNA	Non-Hypermut. proviral DNA	All sequences (OGVs and proviral DNA)					
**CAP188**	247	246	251	5.42	69 (13)	47 (30)	NA (5)	40 (25)	55 (42^)	0	14	T tropic	CCR5	EFV/3TC/TDF EFV/FTC/TDF
**CAP206**	274	286	240	5.30	58 (19)	50 (14*)	33 (6*)	63 (8)	55 (33*)	15	12	T tropic	CCR5	EFV/3TC/TDF EFV/FTC/TDF
**CAP217**	360	239	256	4.90	50 (16)	50 (16)	43 (7)	56 (9)	50 (32)	3	9	T tropic	CCR5	EFV/3TC/TDF
**CAP222**	318	290	305	3.65	NA (2)	55 (11)	NA (1)	50 (10)	50 (12^)	0	8	NA (2)	CCR5	EFV/3TC/TDF EFV/FTC/TDF
**CAP244**	380	268	241	4.40	NA (1)	53 (30)	45 (22)	75 (8)	52 (31)	3	16	NA (1)	CCR5/ CXCR4	EFV/3TC/TDF
**CAP257**	249	318	138	4.72	93 (44)	77 (30)	70 (23)	100 (7)	86 (74)	3	7	T tropic	CCR5/ CXCR4	3TC/NVP/TDF EFV/3TC/TDF EFV/FTC/TDF
**CAP268**	221	380	163	3.56	NA (5)	93 (56)	96 (28)	89 (28)	93 (61)	0	2	NA (5)	CCR5	EFV/3TC/TDF
**CAP277**	258	266	281	3.90	NA (1)	43 (30)	39 (18)	50 (12)	43 (30^)	7	7	NA (1)	CCR5	EFV/3TC/TDF
**CAP280**	300	250	174	4.85	NA (5)	59 (29*)	NA (4)	56 (25*)	56 (34*)	12	6	NA (5)	CCR5	EFV/3TC/TDF
**CAP287**	260	223	216	4.24	67 (6)	53 (30)	52 (25)	NA (5)	56 (36)	19	6	T tropic	CCR5	EFV/FTC/TDF
**CAP288**	210	275	253	4.28	100 (7)	81 (59*)	82 (28*)	81 (31)	83 (65*^)	2	11	T tropic	CCR5	EFV/FTC/TDF
**CAP302**	164	270	204	4.67	17 (6)	30 (46)	29 (31)	33 (15)	29 (52)	29	10	T tropic	CCR5	EFV/3TC/AZT EFV/FTC/TDF
**CAP316**	215	215	278	3.75	92 (13)	73 (26)	NA (4)	77 (22)	79 (38^)	0	3	T tropic	CCR5/ CXCR4	3TC/AZT/Lpvr/r
**CAP333**	193	262	216	4.84	NA (1)	63 (46*)	55 (22*)	71 (24)	62 (47*)	11	4	NA (1)	CCR5	EFV/3TC/TDF EFV/FTC/TDF
**CAP336**	142	258	74	5.42	91 (11)	100 (23)	100 (16)	100 (7)	97 (31^)	0	13	T tropic	CCR5	EFV/3TC/TDF
**CAP337**	166	279	267	4.83	NA (1)	62 (13)	50 (8)	NA (5)	64 (14)	14	7	NA (1)	CCR5/ CXCR4	EFV/3TC/Lpvr/r TDF/3TC/Lvpr/r
**CAP372**	182	247	309	5.22	50 (10)	59 (59)	77 (22)	49 (37)	59 (68^)	1	15	T tropic	CCR5	EFV/FTC/TDF
**CAP380**	133	263	377	4.72	NA (0)	29 (17)	33 (6)	27 (11)	29 (17)	35	12	NA (0)	CCR5	EFV/FTC/TDF
**Median**	**234**	**265**	**246**	**4.72**	**68**	**57**	**51**	**60**	**56**	**3**	**9**			
**Average**	**237**	**269**	**236**	**4.59**	**69**	**60**	**57**	**64**	**61**	**9**	**9**			

* Sequence dating was ambiguous and not included in % Late calculation

^ Identical OGV and DNA sequence considered one sequence

#FPR and coreceptor inhibitor

† Sequences with 4 or fewer differences were collapsed.

The 18 women in this study initiated therapy after an average of 237 weeks (4.6 years; IQR 3.6–5.2 years) of untreated infection (Table 1). After an average of 269 weeks (5.2 years; IQR 4.8–5.3 years) on ART, each participant provided a 200 ml blood sample for isolation of peripheral blood mononuclear cells (PBMCs) for analysis of HIV-1 in the pool of long-lived cells. Viral outgrowth assays were performed using resting CD4+ T cells [32], and wells with outgrowth virus (OGV) (i.e. p24 positive wells) generated at end-point dilution were used to extract RNA to generate cDNA and perform bulk PCR amplification for sequencing. Proviral DNA was examined by first extracting total DNA from PBMCs, and a nested PCR strategy was used to amplify proviral DNA at limiting dilution.

We sequenced the 3’ half of the genome for both proviral DNA and OGVs. Proviral DNA sequences were generated for all 18 women and OGV sequences for 17 of 18 women. Downstream analyses comparing OGVs to proviral DNA focused on the 10 women who had 6 or more unique OGV sequences and 6 or more unique proviral DNA sequences (Table 1). Across participants, an average of 23% of viral sequences (OGV and non-hypermutated proviral DNA) had a 3’ half genome that differed from another viral DNA sequence by 4 or fewer nucleotides. Because it is possible that these variants represent a single entry into the reservoir and subsequent clonal expansion of the cell with that integrant, we collapsed these clonal sequences into a single unique sequence to focus the analysis on independent entry events into the long-lived cell pool.

### Non-hypermutated proviruses and outgrowth viruses (OGVs) are often genetically similar to vRNA circulating near the time of ART initiation

We analyzed a total of 730 unique viral sequences present in cells collected from the participants while on ART, of which 22% were OGVs and 78% were proviral DNA (Fig 1B). Of the 569 unique proviral DNA sequences sampled, 49% were significantly hypermutated as determined by the Hypermut2 algorithm (hiv.lanl.gov). Hypermutations are generated during viral DNA synthesis after infection, when the minus strand of viral DNA is exposed to APOBEC3G/F (A3G/F) and the nucleotide deoxycytidine is deaminated to give deoxyuridine in the minus strand of single-stranded viral DNA [33]. This leads to characteristic deoxyguanosine to deoxyadenosine mutations at the first position of GG and GA doublets in the plus strand of viral DNA. An excess of these changes is likely to be genetically catastrophic, making these mutated DNAs easily recognized as defective proviral DNA. While non-hypermutated proviral DNA sequences lack APOBEC3G/F-mediated mutations, the majority will have other defects that prevent them from producing infectious virus [34], therefore we could not associate the lack of hypermutation with replication-competence or genome intactness given that we sequenced only the 3’ half of the genome.

For each participant, phylogenetic analyses were performed comparing vRNA sequences from multiple timepoints pre-ART to OGV and non-hypermutated proviral DNA sequences (Figs 1C and S3–S20). We initially excluded hypermutated proviral DNA from these analyses due to challenges in interpreting phylogenetic trees that included hypermutated sequences. Our three previously described dating methods [27] were used to generate a consensus time estimate of when each OGV or proviral DNA sequence existed as replicating virus pre-ART and therefore when it infected a cell that contributed to the long-lived infected cell pool. We then determined whether each viral sequence represented a virus circulating in the year before ART (i.e. late) or at a timepoint more than one year before ART (i.e. early). This allowed the viral sequences that were persisting in each participant to be represented by a single value (percent late).

Within each participant, the percent late for OGVs and non-hypermutated proviral DNA (Fig 1D) were highly correlated (Pearson Correlation; R = 0.84, P<0.005). In order to examine whether there was a difference between the percent late for OGVs and non-hypermutated proviral DNA, we examined percent late estimates from the 9 participants with a substantial number of OGV and non-hypermutated proviral DNA sequences (6 or more unique OGVs and unique non-hypermutated proviral DNA sequences) and determined that there was no statistical difference between estimates of percent late generated from these different types of viral sequences (S21A Fig; Wilcoxon Matched Pairs Rank Sum, P = 0.91). In addition, we identified the linear regression that best fits these estimates and determined that the 95% confidence interval around this line includes the line predicted if the percent late were the same for OGVs and non-hypermutated proviral DNA (Fig 1D). Together these analyses indicate that within each participant, the percent late was similar for OGVs and non-hypermutated proviral DNA. While the percent late is highly variable across participants, the majority of both the OGVs and non-hypermutated proviral DNA sequences entered the long-lived infected cell pool at timepoints in the year prior to ART initiation (an average percent late of 69% and 60%, respectively, Table 1).

### Timing of entry of hypermutated proviral DNA into the long-lived infected cell pool

**Fig 2:**
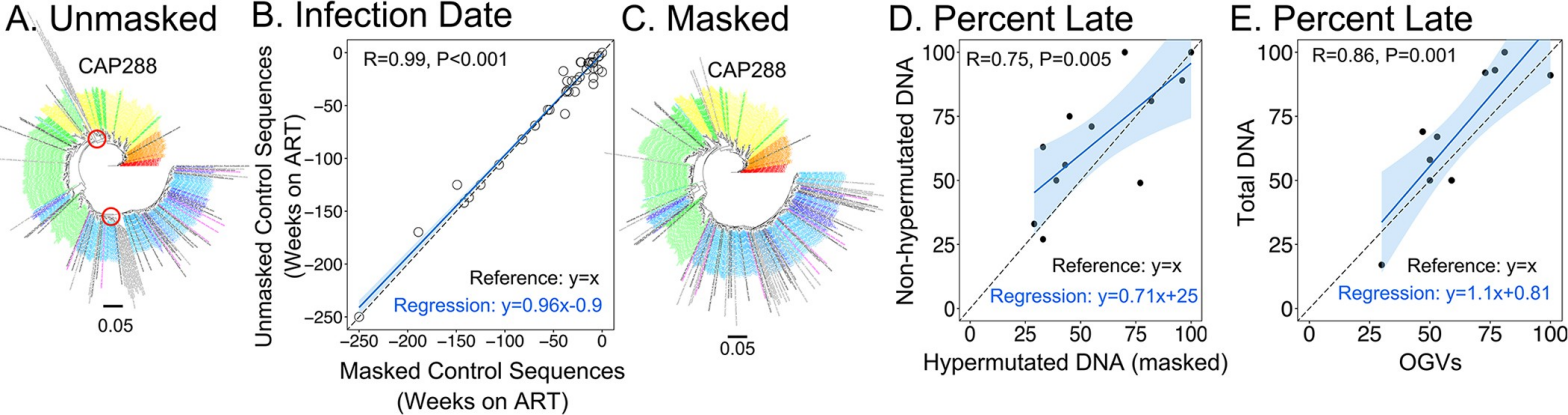
Dating the Entry of Hypermutated Proviral DNA Into the Pool of Long-Lived Cells. **A.** The phylogenetic tree of the sequences for CAP288 as described in Fig 1 but now including the hypermutated proviral DNA sequences (with gray labels) which cluster in branches indicated with the red circles and preclude dating of long-lived sequences. **B**. The graph shows the data comparing the dating of OGV sequences with the same sequences where there has been masking of potential APOBEC3G/F target sequences (the first G of GG and GA doublets as represented in the plus strand). The dating of each sequence assessed with and without masking is shown as time before ART initiation (negative Weeks on ART) as an individual point. The similarity of the entry time into the pool of long-lived cells assessed with and without masking is shown by the strong correlation (R = 0.98, P<0.001). **C.** The phylogenetic tree of the sequences from CAP288 shown in panel A is now presented with the hypermutated sequences having sites with evidence of APOBEC3G/F mutation masked, with the gray sequences now distributed in the tree. **D**. The percentage of late viral sequences identified in the non-hypermutated and the hypermutated (masked) forms of the long-lived viral sequences are compared to each other for each of twelve participants. This analysis was limited to the participants who had at least six unique sequences in each of these groups of proviral DNA. There is significant correlation between the two types of viral sequences (Pearson; R = 0.75, P = 0.005) and the 95% confidence interval around the linear regression that best fits the data includes the line that would be expected if both forms of viral DNA had equal percentages of their sequences derived from late replicating viruses (y = x). **E**. The same approach was used to examine the percentage of late viral sequence identified in proviral DNA (both masked hypermutated and non-hypermutated) and OGV in nine participants. The association between percent late estimates generated from total proviral DNA and OGVs was within the 95% confidence interval of the observed relationship.

We were interested in developing a method that allowed us to determine the timing of entry of defective, hypermutated proviral DNAs into the long-lived pool of infected cells. However, a limitation of our timing approach is that the presence of hypermutations caused these sequences to cluster together in the phylogenetic tree (See Fig 2A for sequences from CAP288, lineages with red circles and gray sequence labels), which precluded the use of the longitudinal sampling of vRNA from the pre-ART replicating virus to infer when these sequences entered the pool of long-lived infected cells. To overcome this problem, we developed an algorithm to mask the target position in the GG and GA doublets, thus masking the putative hypermutated positions if present. One concern was that by reducing the number of bases being analyzed, masking could impair our ability to accurately estimate when a sequence entered the long-lived pool of infected cells. In order to validate this approach, we took replication competent OGVs that lacked hypermutations and estimated when they entered the inducible reservoir that persisted in long-lived cells. We then masked the sites susceptible to hypermutation and estimated entry into the reservoir. As can be seen in Fig 2B, the masking of potentially hypermutated sites did not impair our ability to estimate when control sequences (i.e. OGV sequences) entered the reservoir, and the method generated estimates that were not significantly different from those generated by the unmasked control sequences (S22 Fig; Wilcoxon Rank Sum; P = 0.68). We then applied this approach to hypermutated proviral DNA sequences from one participant and found that once masked, the hypermutated sequences no longer clustered but were distributed throughout the phylogenetic tree (compare Fig 2A to 2C).

Next, we masked hypermutated positions in all hypermutated proviral DNA sequences (S1 Table) and examined when the hypermutated proviral DNA sequences entered the pool of long-lived cells using the masked hypermutated sequences in the analysis (Phylogenetic trees shown in S3–S20 Fig). As was done in Fig 1D, the timing of infection was summarized as the percent of the sequences similar to viruses replicating in the year before ART initiation (i.e. percent late). The percent of masked hypermutated proviral DNA sequences that entered the long-lived cell pool late was highly correlated with the percent of non-hypermutated proviral DNA sequences that entered late (Fig 2D; Pearson; R = 0.75 and P = 0.005), and there was no statistical difference between these estimates (S21B Fig; Wilcoxon Rank Sum; P = 0.14). This suggests that the same mechanisms that determine when non-hypermutated proviral DNA sequences enter the long-lived cell pool appear to also govern the timing of entry of hypermutated proviruses into the long-lived cell pool.

### Within participants, both replication-competent and putative defective sequences are seeded into the long-lived pool of infected cells at similar times

We observed that the percent of OGVs and total proviral DNA (non-hypermutated and hypermutated) sequences corresponding to variants circulating in the year before ART initiation were highly correlated (Fig 2E; Pearson; R = 0.86, P = 0.001) and not statistically different (S21C Fig; Wilcoxon Rank Sum; P = 0.12).

To investigate if the OGVs were preferentially eliminated over time from the pool of long-lived cells, we compared the ratio of OGV viruses and proviral DNA that entered the long-lived pool late near the time of ART versus the ratio that entered earlier in infection. For nine of ten participants with 6 or more OGVs, we did not detect an association between the type of viral sequence (OGV or proviral DNA) and when (early or late) it entered the long-lived cell pool (Fisher’s Exact Test, p>0.05, S2 Table), indicating that OGVs are not underrepresented in the early reservoir relative to other forms of viral sequences in the long-lived pool. For the one participant (CAP 336) with a significant association, we did not identify any OGV sequences seeded early. For this individual, OGVs were underrepresented among viral sequences seeded early in untreated infection; however, this result was not statistically significant when corrected for multiple comparisons (See S2 Table).

### Timing of the introduction of HIV-1 sequences into the long-lived infected cell pool across the cohort

**Fig 3:**
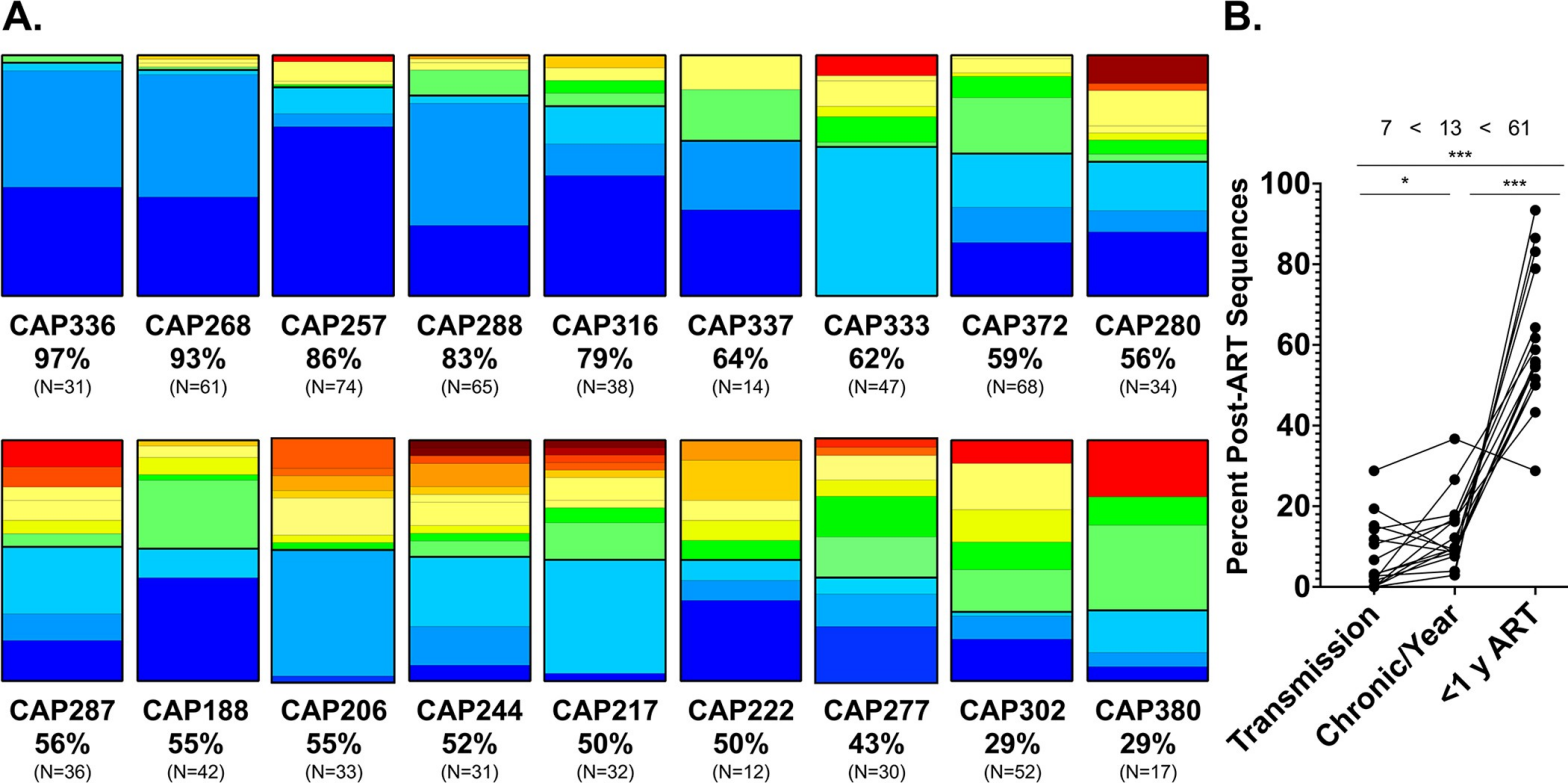
The Distribution of the Times Viral Sequences Entered the Pool of Long-Lived Cells Across the Cohort. **A.** Each bar graph represents the distribution of entry into the pool of long-lived cells for an individual participant; the analysis pooled all unique sequences from OGVs, non-hypermutated proviral DNA, and hypermutated proviral DNA (masked). The total number of viral sequences analyzed is shown below each graph, along with the percent of sequences that cluster with variants circulating in the year before ART (percent late). The color scheme is the same as in Fig 1B with blue shades representing the year just before the initiation of therapy, red representing the first year after diagnosis of infection, and orange, yellow, and green representing intervening years. A black line is included to indicate the break between the late viruses (last year before therapy) and the earlier viruses. The bar graphs are ordered from the highest percentage of late sequences to the lowest percentage. **B.** The fraction of the viral sequences (estimated from both OGV and proviral DNA sequences) that entered the pool of long-lived cells each year for each participant is shown, where each line is for an individual participant. The fraction that entered during the first year after transmission is shown on the left, the fraction that entered in the last year prior to the initiation of therapy is shown on the right, and the point in between represents the fraction that entered on a per year basis between the first year of infection and the last year before therapy. The average value for each of these time frames is shown at the top of the graph (7% in the first year, 61% in the year just before therapy initiation, and 13% per year between the first and last years prior to therapy). The bars at the top indicate comparisons with * representing a P value of <0.05 and *** representing a P value of <0.001 as assessed using a Wilcoxon Matched Pairs Rank Sum Test.

In the previous analyses (Figs 1 and 2) we did not detect a difference in when OGV viruses, non-hypermutated proviruses and hypermutated proviruses enter the pool of long-lived cells. To increase the sample size characterizing the timing of entry, we next generated an assessment of the timing of entry of all viral sequences across the group of 18 participants. For this analysis we pooled all viral sequences from the three different sources (OGV, non-hypermutated proviral DNA, masked hypermutated proviral DNA). Timing of entry into the long-lived pool of cells is shown as a bar graph for each participant (Fig 3A), with blue colors representing viruses replicating within the last year before therapy and the red colors representing viruses replicating in the first year of infection (color scheme shown in Fig 1C). The percentage of late virus is shown below each bar. As can be seen in Fig 3A, there is a wide range of the percentage of late virus in the pool of long-lived cells among the participants. On average, 61% of the persistent viral sequences are present in cells infected in the year before ART initiation which is slightly lower than our previous estimate from a subset of this group and based only on examining OGV sequences (70%; [27]). In 83% of the participants, at least 50% of the viral sequences that persisted on therapy were composed of viruses replicating within the last year before therapy.

Primary infection represents a unique time with high viremia only partially controlled by the adaptive immune response. We were interested in seeing if, across participants, viruses from near the time of transmission were disproportionately represented as viral sequences in the long-lived pool of infected cells. For each participant we plotted the fraction of the viral sequences in long-lived cells that represented virus replicating in the first year of infection, the fraction of the viral sequences that represented virus replicating in the last year of infection, and the per-year rate of virus entering the pool of long-lived cells between the first and the last year of untreated infection. Two of the participants (CAP336 and CAP380) were excluded from this analysis because they were untreated for less than 3 years. As can be seen in Fig 3B, the accumulation of sequences into the pool of long-lived cells in the first year (i.e. the year after transmission) was slightly lower than the per-year rate of accumulation of sequences into the pool of long-lived cells prior to the final year before therapy (7 vs 13% per year, Wilcoxon Matched-Pairs Rank Sum test, P = 0.02). In contrast, entry into the pool of long-lived cells in the year before therapy (i.e. late) was much higher than in the first year of infection (P <0.0001) and during the intermediate years (P <0.0001). Thus, while virus replicating soon after transmission can contribute to the viral sequences in the pool of long-lived cells, it does not do so at an enhanced rate in this cohort of people initiating therapy during chronic infection.

As noted above, these analyses were of unique sequences to allow us to estimate entry into the pool of long-lived cells. This is distinct from the frequency of a sequence in this pool which is impacted by clonal proliferation of cells. Hence, we next performed an analysis to determine whether viral sequences that entered the long-lived pool of cells early (more than a year before therapy initiation) or late (within the year before therapy initiation) were more likely to be detected as part of a large, expanded clone. For this analysis we identified 7 participants whose pool of viral sequences in the long-lived cells contained 40%-60% unique late sequences so that both early and late viral sequences would be well represented. To make it equally likely to identify clones in these participants, we randomly sampled 32 proviral DNA sequences per participant (without replacement; OGV sequences were not used in this analysis) and combined these sequences (N = 224 total sequences). We then collapsed the dataset to unique sequences (N = 181 unique sequences) noting which unique sequences were part of a clone. We found that the percent of unique proviral DNA sequences that represented a clone was similar for early (12.9%) and late (8.3%) entering sequences (Fisher’s Exact Test, P = 0.34).

### Similar frequency of CXCR4-using viruses in the pool of long-lived cells as those circulating at the time of ART

**Fig 4:**
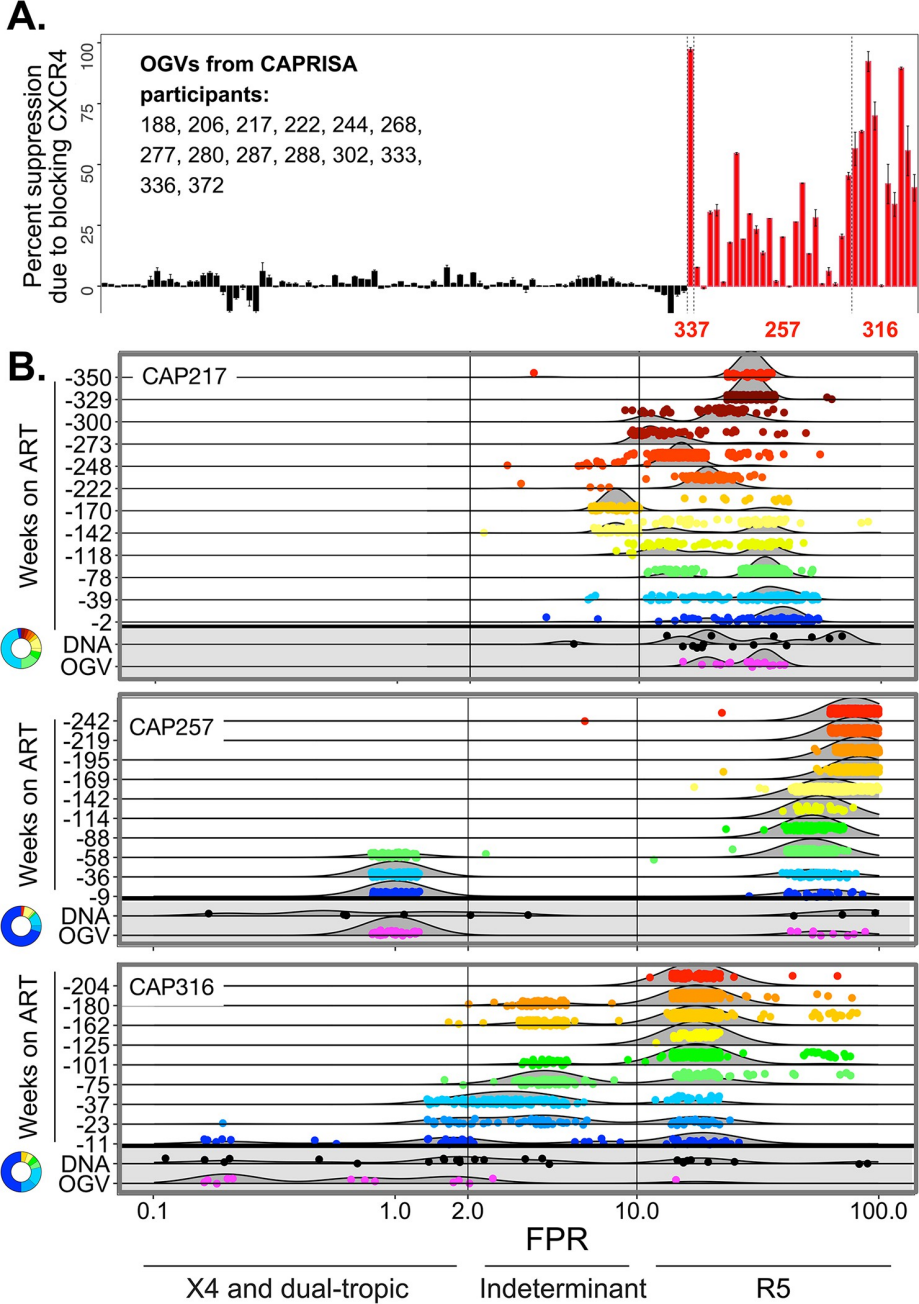
Entry of X4 Viruses Into the Pool of Long-Lived Cells. **A.** OGVs were tested for use of CCR5 and CXCR4 for coreceptor as a function of their sensitivity to maraviroc and AMD-3100, respectively. The graph shows the amount of viral infectivity for each OGV that was blocked by AMD-3100, indicating dependence on CXCR4 for infectivity. OGVs from CAP257 and CAP 316 showed a high percentage of viruses that required CXCR4 for efficient entry, with OGVs from other participants showing no ability to use CXCR4 and only CCR5 usage. **B.** All viral sequences (from pretherapy replicating virus and from proviral DNA and OGVs in the pool of long-lived cells) were plotted as a function of their False Positive Rate (FPR) value as determined based on the *env* V3 sequence analyzed using Geno2Pheno (https://coreceptor.geno2pheno.org/). The horizontal lines represent individual time points of replicating viral sequences (negative values for Weeks on ART) with the FPR value for each sequence color-coded for time (as in Fig 1), or viral sequences from the pool of long-lived cells as either proviral DNA (black dots) or OGVs (as magenta dots). CAP217 is shown as a control who did not develop X4 viruses prior to the initiation of therapy. CAP257 and CAP316 developed X4 viruses close to the time of therapy initiation. FPR values of less than 2 are considered reliably identified as being able to use CXCR4 for entry, with some viruses retaining the ability to still enter using CCR5 (termed dual-tropic) while some have lost the ability to use CCR5 (pure X4). FPR values greater than 10 are considered to reliably identify viruses that only use CCR5. FPR values between 2 and 10 are considered indeterminant in that they represent some viruses that can use CXCR4 and others that use only CCR5 making phenotypic calls for viruses with this range of FPR values unreliable. The graphs show that viruses that were circulating at the time of therapy initiation entered the pool of long-lived cells regardless of entry phenotype.

The enhanced probability of entry into the pool of long-lived cells within the last year before therapy can also be seen in the entry phenotype encoded in the viral sequences. HIV-1 typically uses CCR5 as the coreceptor with CXCR4-usage occasionally evolving late in the course of infection (reviewed by [35]). We previously found [36] that an algorithm for predicting coreceptor usage (Geno2Pheno [37]) accurately predicts CXCR4 usage (X4 virus) when it calculates a False Positive Rate (FPR) less than 2%, but is only about 50% accurate for viruses with FPR values between 2% and 10%; in contrast, FPR values greater than 10% are a reliable predictor of CCR5-usage (R5 viruses) [36]. OGVs from this cohort were experimentally tested for the ability to use CXCR4 for entry (Fig 4A) and this revealed four participants with X4 viruses in their reservoir of inducible virus, with two having a high percent of X4 variants (CAP257 and CAP316). In Fig 4B we show that when viral variants with small FPR values (predicted X4 variants) evolved late in untreated infection, they were equivalently represented in OGVs and total proviral DNA in the pool of long-lived cells. Consistent with previous studies [24], X4 entry phenotype is not a barrier to entry into the pool of long-lived cells.

In addition, we examined whether the phenotype encoded in the viral sequences in the pool of long-lived cells, like viruses typically found in the blood throughout untreated infection, are adapted to entering CD4+ T cells and not macrophage (i.e. are T cell-tropic) [38]. Macrophage-tropism was assessed by determining whether OGVs (supernatant from outgrowth wells or pseudotyped viruses generated using cloned *env* genes) have an enhanced sensitivity to sCD4, which is a strong predictor of M-tropism [39,40]. We observed that all OGVs (N = 126 total OGVs) from the ten participants with at least 6 OGVs (Table 1) were relatively resistant to sCD4 compared to the M-tropic virus controls indicating that all OGVs were T cell-tropic.

### Late entry of virus into the long-lived cell pool is correlated with nadir CD4+ T cell levels

**Fig 5:**
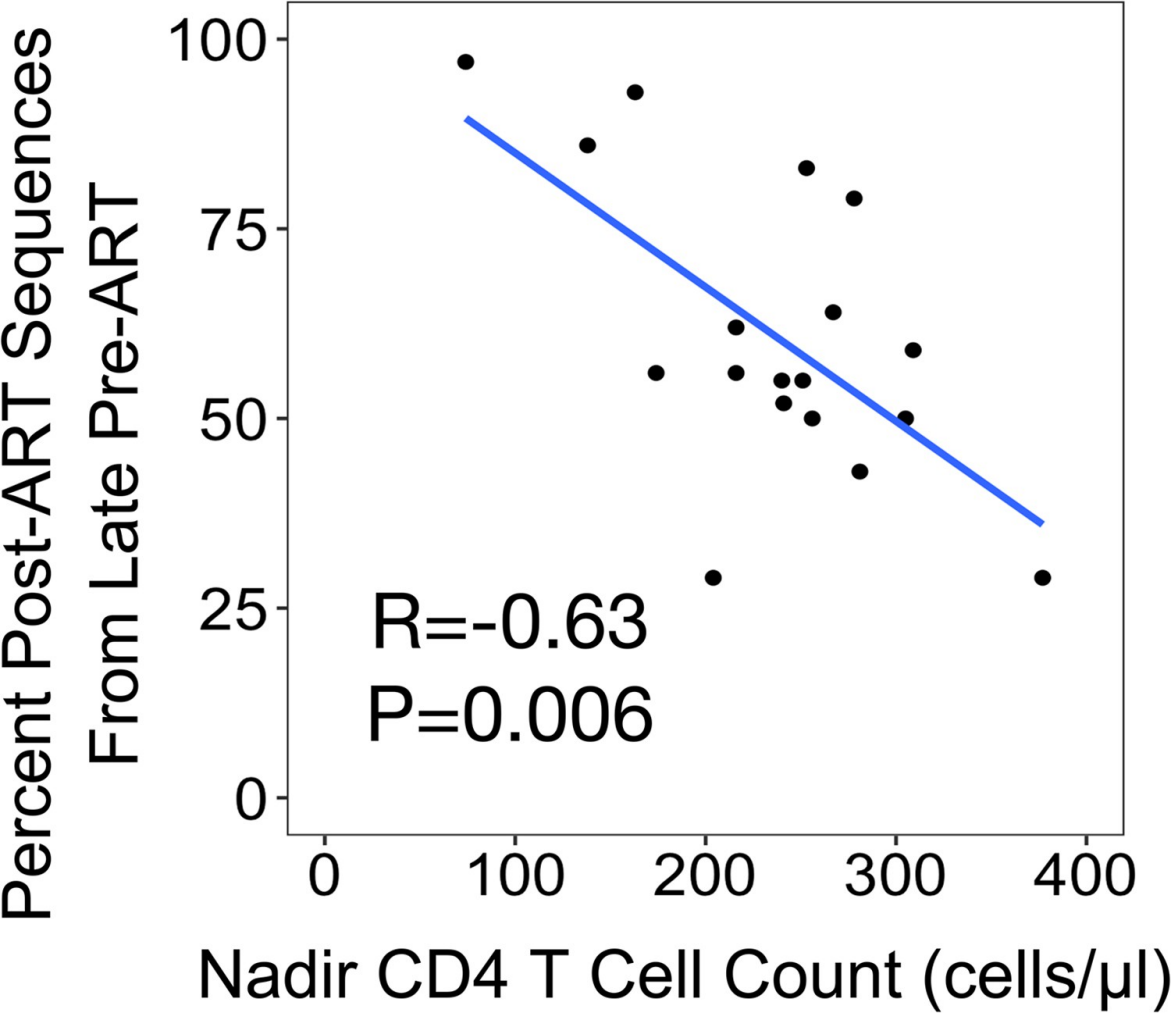
Entry of Viral Sequences Into the Long-Lived Pool of Cells Near the Time of Therapy Initiation ("Late") Is Correlated With Low Nadir CD4+ T Cell Counts. The percentage of unique post-ART sequences (OGV and proviral DNA) representing late entry into the pool of long-lived cells (i.e. viral sequences that were replicating within the year before the initiation of therapy) for each participant (Fig 3) is plotted as a function of their nadir CD4+ T cell count prior to the initiation of therapy. As shown in the figure, there is a significant, inverse correlation (Pearson; R = -0.63, P = 0.006), with lower nadir CD4+ T cell counts being associated with a higher percentage of late viral sequences in the pool of long-lived cells.

We observed that the timing of the entry of viral sequences into the long-lived pool of cells varies greatly across participants (Fig 3A) with some participants having viral sequences almost completely derived from late replicating viruses (e.g. CAP336 at 97%) to the other extreme where a minority of viral sequences came from late replicating viruses (e.g. CAP380 at 29%). This wide range in the composition of viral sequences in the long-lived cell pool suggests there are important biological variables that impact this distribution. We considered CD4+ T cell counts as one variable that might affect how efficiently sequences from replicating virus would enter the pool of long-lived cells. Nadir CD4+ T cells counts can be used as a surrogate of immunodeficiency, and their loss leads to dysregulation of T cell homeostasis. We found a significant negative correlation between the percentage of viral sequences in the long-lived cell pool that were seeded in the year before ART initiation (i.e. late) and nadir CD4+ T cell counts (Fig 5; Pearson; R = -0.63, P = 0.006) indicating that lower CD4+ T cell counts are associated with a higher percentage of viral sequences being seeded near the time of ART initiation. It is worth noting that due to treatment guidelines in place during enrollment, individuals in this cohort had relatively uniform nadir CD4+ T cell counts that were lower than observed in many people initiating ART during the subsequent ‘test and treat’ era.

## Discussion

A detailed understanding of the dynamics of HIV-1 reservoir formation is imperative to designing strategies to limit or eliminate the source of viral rebound following treatment interruption. Here, in the first study directly comparing when inducible replication competent and the largely defective proviruses entered the long-lived pool of cells that persist on therapy, we observed four primary patterns. First, both inducible replication competent and largely defective proviruses in long-lived cells are composed mainly of cells infected near the time of ART initiation. Second, both proviral DNA of various forms and replication competent variants (i.e. OGVs) entered the pool of long-lived cells at similar proportions early and late in untreated infection. Third, lower nadir CD4+ T cell counts were associated with a higher proportion of the long-lived infected cells becoming infected near the time of ART initiation. Fourth, the cellular tropism of variants in the long-lived cell pool largely reflects the tropism of variants circulating near the time of ART initiation. Together, our results are consistent with enhanced seeding of long-lived infected cells near the time of ART initiation being driven by changes in CD4+ T cell survival and/or proliferation, and not by replication competency or co-receptor usage of the provirus.

This study aimed to better understand when all forms of viral DNA enter the pool of long-lived cells and how that process is impacted by replication competency. Given the large variation in the timing of reservoir seeding across participants [27,30], studies examining how replication competency impacts the timing of reservoir formation must examine replication competent and defective proviruses in the same participants. In this study, we build on our previous work examining the timing of reservoir formation by not only expanding our cohort from 9 to 18 women, but also comparing when inducible replication competent (OGVs) and the defective proviruses enter the pool of long-lived cells in the same participants. Here we utilized samples collected every 6 months from women who enrolled in the CAPRISA 002 study soon after HIV-1 infection, remained untreated for at least 2.5 years, initiated ART based on prevailing country guidelines and remained well-suppressed for more than 4 years prior to analysis of the viral sequences in the long-lived cell pool. We find that inducible replication competent OGVs and largely defective proviral DNA sequences preferentially enter the pool of long-lived cells in the time around ART initiation (69% and 60%, respectively). This is consistent with our previous study of replication competent proviruses [27] and several studies of proviral DNA [28–30,41] which also found that variants replicating around the time of ART initiation make up the bulk of the viral sequences in the pool of long-lived infected cells in the blood. The most important part of the viral sequences in the long-lived pool of cells is the fraction of intact proviruses that can rebound if therapy is discontinued. However, given that all of the forms of the long-lived viral sequences that we characterized enter the long-lived cells at similar times, we can infer that the portion of the reservoir capable of rebound enters the long-lived cells with similar timing, although host effects may limit which viruses ultimately rebound [42].

To explore whether replication competent variants that enter the pool of long-lived cells early in untreated infection are cleared more efficiently during untreated infection than defective proviruses, we examined whether replication competent variants seeded early are underrepresented in the long-lived pool relative to total proviruses (most of which are defective) seeded early. There was no significant association between the timing of reservoir seeding and whether the virus was replication competent. This suggests that selection during untreated infection does not remove replication competent variants from the long-lived cell pool much more efficiently than defective variants. While it is possible that replication competency has a small impact on viral persistence before ART, the massive loss of both uninfected and infected CD4+ T cells during untreated infection [43] suggests that virus production is not the primary factor causing depletion of CD4+ T cells before ART. In contrast, there is substantial evidence that immune selection during ART targets cells harboring replication competent proviruses more than those with defective proviruses. For example, after initiation of ART, intact proviruses decay more rapidly than defective proviruses [44]; presumably because they express more viral proteins and/or infectious virus. In addition, a recent study of PWH observed that after many years of ART (18–23 years) intact, but not defective, proviruses become enriched in non-genic regions [45]. Together these observations suggest that while replication competency has little influence on when variants enter the pool of long-lived cells prior to ART, intact proviruses are subject to greater immune selection during ART. In this regard we did detect a small (but not statistically significant) increase in the percent of the viral sequences introduced late defined as OGVs (69%) compared to non-hypermutated proviral DNA (60%), a difference that is consistent with early OGV sequences being slightly underrepresented.

This, and other studies [27, 30], have observed that when viral sequences enter the long-lived pool of infected cells (measured as the percent that enters in the year before ART or percent late) varies greatly across participants (range 29–97% late in this study). Our findings also revealed an association between a lower nadir CD4+ T cell count and a higher fraction of viral sequences being seeded near the time of ART initiation. Based on work showing that initiation of ART increases the average half-life of CD4+ T cells [46, 47], we previously modeled the relationship between cellular half-life before ART and the timing of reservoir formation [27]. This model revealed that more rapid turnover of HIV-infected cells prior to ART increases the proportion of the reservoir that forms near the time of ART initiation [27]. It is possible that conditions that generate greater CD4+ T cell depletion during untreated infection (i.e. lower nadir CD4+ T cell counts) also result in more rapid turnover of HIV-infected cells, thus reducing the probability that cells infected early in untreated infection contribute to the pool of long-lived infected cells. The factors controlling cellular half-life before and after ART initiation are not understood, but the increase in half-life after ART initiation may stem from the fact that ART-mediated viral suppression restores the ability of cells to transition to a long-lived memory phenotype [27,48], protects cells from superinfection and/or reduces immune system-activating inflammation.

Homeostatic proliferation of HIV-infected cells may also drive the association between viral sequences being preferentially seeded near the time of ART initiation and the immunologic state of the host. After ART initiation, homeostatic proliferation contributes to immune reconstitution of and increases in the number of CD4+ T cells. Homeostatic proliferation of recently infected cells after ART initiation could increase the frequency of late viral variants in the pool of long-lived cells. Lower CD4+ T cell counts in people living with HIV-1 and on ART are associated with an increased frequency of CD4+ T cells expressing Ki67 [49], a marker of immune activation and cellular proliferation. Similarly, a nonhuman primate study observed that homeostatic proliferation increased after antibody-mediated depletion of CD4+ T cells [49]. Extensive proliferation of HIV-infected cells may also explain the observation that we [50] and others [51] have made that nadir CD4+ T cell count is negatively associated with reservoir size. Additional analyses are needed to determine whether more rapid turnover of infected cells prior to ART, elevated homeostatic proliferation at the time of ART initiation and/or some other factor, drive the negative association between nadir CD4+ T cell count and the proportion of the viral sequences that is seeded near the time of ART initiation. Conversely, while viral sequences can enter the pool of long-lived cells at any time during the untreated infection, we found that the first year after infection is on average the least active time of entry into this pool of cells.

Here we performed the first study examining whether tropism impacts the timing of entry into the pool of long-lived cells. Viruses circulating during untreated infection are almost exclusively well-adapted to infection of CD4+ T cells and not macrophages (i.e. are T cell-tropic, requiring a high density of CD4 for efficient entry) [38]. All viral sequences in the long-lived cell pool that we examined were largely resistant to sCD4, indicating that viruses seeded into long-lived cells at different times during untreated infection are typically T cell-tropic. This is in contrast to a recent study that found a minority of rebound viruses were adapted to infecting macrophage [52]. We also performed entry assays to assess coreceptor-usage of OGVs and found that the majority were CCR5-using, but four participants had CXCR4-using OGVs. We then examined *env* sequences (OGVs and proviral DNA) and observed that the ratio of CCR5 to CXCR4 coreceptor-using viruses in the pool of long-lived cells was similar to the ratio co-circulating at the time of ART initiation, suggesting that CXCR4-using variants are not restricted from entering the pool of long-lived cells [24].

A better understanding of the factors shaping the entry of viral sequences into the pool of long-lived cells presents opportunities for interventions that exploit these factors. Our work suggests that interventions at the time of ART initiation may present the best opportunities for reducing the size of the reservoir by preventing recently infected cells from entering the reservoir. Further, interventions to augment antiviral immunity, particularly early in ART should consider the risk of inducing T cell proliferation, with attendant expansion of HIV-infected cells. Additional work is needed to identify effective interventions and examine their impact on viruses replicating both early and late entering the reservoir.

## Materials and methods

### Ethics statement

This study was reviewed and approved by University of Cape Town Institutional Review Board (IRB), the University of KwaZulu-Natal IRB and UNC. Written informed consent was obtained from all participants.

### Study participants

Women from rural and urban KwaZulu-Natal, South Africa, were enrolled in the CAPRISA 002 cohort and identified during the period of acute/primary infection with subtype C HIV-1 and followed longitudinally.

### Pre-ART viral RNA sequencing

We used our previously described MiSeq with Primer ID approach to sequence HIV-1 RNA isolated from longitudinal plasma samples collected before ART [27]. We sequenced the following five regions of the HIV-1 genome: ENV2 (C1C2, HXB2 #6585–6950), ENV3 (C2C3, HXB2 #6839–7321), ENV4 (C4C5, HXB2 #7371–7685), NEF (HXB2 #8786–9201) and GAG (p17, HXB2 #814–1281) when available. These regions were selected because they showed the most consistent increase in sequence diversity over time, which was quantified by generating a consensus sequence from the earliest timepoint (LANL Simple Consensus Maker) and measuring the average pairwise diversity to that consensus for sequences at each timepoint (MEGA 7.0.26). The slope of the average pairwise diversity over time was highest in these regions, as shown in S1 Fig.

### Viral outgrowth assay

The viral outgrowth approach was described in our previous study [27]. Briefly, negative selection was performed on PBMCs to isolate resting CD4+ T cells that were CD69-, HLA-DR-, CD25low (Custom kit, Stem Cell). Purity was assessed by flow cytometry and the data was analyzed with FlowJo (version 10.4.2). Cells were cultured at 100,000 cells/well with highly purified phytohemagglutinin, interleukin 2, and irradiated PBMCs from a seronegative donor, and our previous method was used to facilitate outgrowth [32]. Culture supernatants positive for p24 were identified on days 15 and 19, and the virus-positive supernatants were stored at -80°C.

Viral RNA was isolated from the culture supernatant of p24-positive wells and converted to cDNA using SuperScript III Reverse Transcriptase and an oligo(dT) primer. The 5′ and 3’ half genomes were amplified in PCR reactions using barcoded primers, and the PCR products were gel purified.

### Droplet digital PCR

Total DNA was isolated from PBMCs using the DNeasy Blood and Tissue Kit (Qiagen) according to manufacturer’s instructions. Purified DNA was eluted in DEPC-treated water (Ambion). The concentration of HIV-1 DNA in each sample was determined using droplet digital PCR (ddPCR) with primers and probes corresponding to the region spanning the end of the 5’ LTR and the start of *gag* (HXB2 coordinates 684–810) [53]. Cell concentration was determined based on a separate ddPCR reaction targeting the cellular gene *RPP30* [53]. See S3 Table for primer/probe sequences. ddPCR reactions included Supermix for Probes (no dUTP) (Bio-Rad), primers, probes and DNA no-template controls were included in every run. DNA from the 8E5 cell line (containing a defective copy of HIV-1 DNA) was used as a positive control [54]. All reactions were run in duplicate. Droplets were generated using QX200 Automated Droplet Generator (Bio-Rad), and thermal cycling was performed using a C1000 Touch Thermal Cycler (Bio-Rad). Plates were read on a QX200 Droplet Reader (Bio-Rad).

### Half-genome amplification (HIV-1 proviral DNA)

A near full length proviral DNA amplicon was generated in the first round of PCR done at template limiting dilution, followed by 3’ half genome amplification from HIV-1 proviral DNA in the second round of PCR (both the 5’ and 3’ half genomes were amplified and sequenced for OGVs). A nested PCR strategy was used to generate 3’ half genome HIV-1 amplicons from PBMC DNA, with the round two amplicon corresponding to HXB2 coordinates 4653–9632. All primer sequences are in S3 Table. PCR was performed using Platinum *Taq* DNA Polymerase High Fidelity (Thermo Fisher Scientific). HIV-1 proviral DNA was added such that each reaction contained on average one copy of HIV-1 proviral DNA, based on ddPCR estimates; this level of proviral DNA still gave rise to rates of PCR products that indicated amplification was occurring at end-point dilution. A no-template control was included on each plate. To facilitate multiplexing, PacBio barcodes were added to the second-round forward and reverse primers (4653F and OFM19, respectively). Second-round PCR products were analyzed on a 0.8% agarose gel and visualized with a UV gel imager.

### PacBio library construction

The SMRTbell Template Prep Kit 1.0 (Pacific Biosciences) was used to add adaptors to pooled, barcoded amplicons (from either OGVs or proviral DNA), and the amplicons libraries were then submitted for PacBio sequencing (movie time of 10 hours for data collection). Sequences were grouped by barcode and analyzed using the PacBio Long Amplicon Analysis package. The 3′ amplicons were visually screened to confirm that open reading frames were intact. The on-ART viral sequences are deposited in GenBank (accession nos. MN097551–MN097697 and OQ551935–OQ552532) and pre-ART RNA sequences are deposited in Sequence Read Archive (SRA; accession no. PRJNA1034561 and PRJNA550394).

### Analysis of HIV-1 proviral DNA sequences

3’ half HIV-1 proviral DNA sequences from each participant were aligned using MUSCLE 3.8.425 run via Geneious Prime to the corresponding OGV consensus sequence. Next, if a barcode was associated with multiple sequences that differed by 5 or fewer nucleotides, sequences that differed from the most abundant sequence were discarded and the most abundant sequence was collapsed. Sequences with different barcodes that appeared clonal on the tree were aligned and the number of nucleotide differences was enumerated. If the suspected clonal sequences had fewer than 5 nucleotide differences, the sequences were collapsed into a consensus sequence for all downstream analyses. Next, the hypervariable loops in *env* were removed. Potentially hypermutated HIV-1 proviral DNA sequences were identified using Hypermut 2.0 (hiv.lanl.gov) using the participant-specific, OGV-derived consensus sequence as the reference. Sequences that were identified by Hypermut 2.0 as potentially hypermutated were further processed using an in-house Ruby script to mask hypermutated positions (i.e.: the hypermutated positions were changed to ‘N’). Finally, masked hypermutated proviral DNA sequences and non-hypermutated proviral DNA sequences were trimmed into regions corresponding to the longitudinal RNA MiSeq sequences.

As in our previous study [27], three methods (distance, clade support, and phylogenetic placement) were used to analyze each alignment and estimate the pre-ART timepoint when each long-lived viral sequence was circulating. For most participants, proviral DNA sequences were analyzed using four alignments, one for each amplicon in the 3’ half of the genome (Phylogenetic trees shown in S3–S20 Fig). Analyses of OGVs included an additional amplicon in the 5’ half genome (GAG1) that was not used to analyze DNA. The three methods were used to separately analyze each amplicon. For each proviral DNA sequence there were up to 12 estimates of when it entered the vDNA reservoir (4 amplicons/regions each assessed by 3 methods) and for each OGV sequence there were up to 15 estimates (5 amplicons/regions each assessed by 3 methods). A weighted median was used to calculate the consensus estimate of when each sequence entered the pool of long-lived cells.

### Coreceptor tropism assay

Outgrowth virus supernatants were titered on TZM-bl cells. A total of 2x10^4^ TZM-bl cells were plated per well in a 96 well plate; virus-containing supernatants were serially diluted, added and spinoculated for 2 hrs at 2000 rpm (849 *x g*) and 37°C onto the TZM-bl cells. Plates were incubated at 37°C for 48 hr, and then collected and assayed for induction of expression of firefly luciferase. OGV supernatants with sufficient titers were used for the coreceptor tropism assay. Cells were plated as above with no drug, 20 nM, or 2000 nM concentrations of AMD3100 (Millipore-Sigma A5602), Maraviroc (AIDS Reagent ARP-11580), or both, in triplicate in 96 well plates. An amount of virus that would give 250,000 RLUs was added to each well, and plates were spinoculated, incubated and read as above. Pseudoviruses were used as controls and were created by transfecting previously described *env* expression vectors [55] and a Subtype C viral DNA backbone in 293T cells with Fugene HD. Cells were washed once at 24 hrs. Viruses were collected at 48 hrs and passed through a 23 micron filter then stored at -80°C until use in the coreceptor tropism assay.

### Macrophage tropism assay

TZM-bl cells were plated for infection as above in 96 well plates, and 250,000 RLUs of virus were mixed with 0 μg/mL, 2 μg/mL, or 20 μg/mL of sCD4 (Fisher 50-162-4255) for 1 hour at 37°C. Virus and sCD4 were then added to the plate in triplicate and spinoculated as above. Plates were incubated for 48 hrs and read for RLUs as a measure of infection.

## Supporting information

S1 Table Masking potential APOBEC sites. (DOCX)

S2 Table Analysis to determine if OGVs were preferentially eliminated over time from the pool of long-lived cells. (DOCX)

S3 Table Primer Sequences. (DOCX)

S1 Fig Analysis of amplicon sequence quality for estimating time since infection. Thirteen amplicons distributed across the HIV-1 genome were used to analyze RNA genomes isolated from the plasma of 18 participants at an average of 10 pre-ART timepoints. For each amplicon the average pairwise distance (PWD) was calculated between sequences at that timepoint and a consensus from the first timepoint. This was repeated at each timepoint during untreated infection and the relationship between change in PWD and weeks between timepoints was analyzed. For each amplicon, we show the average increase in pairwise distance per year and the correlation between change in PWD and time.
(TIF)

S2 Fig Analyses of sequence date with and without sequences from the 5’ half genome. The addition of a gag amplicon in the 5’ half genome did not have a major impact on the estimated date of individual **A.** OGV or **B.** proviruses. Each circle represents a sequence in long-lived cells and colors correspond to different participants.
(TIF)

S3 Fig Timing of reservoir formation for Participant CAP188. Approximately Maximum-Likelihood trees were used for each of the gene regions; OGV sequences are shown in magenta and proviral sequences are shown in black (non-hypermutated viral DNA) and gray (hypermutated viral DNA). Sequences generated from plasma collected within the first year of diagnosis are shown in shades of red, within the last year before therapy initiation are shown in shades of blue, with times between the first and last year shown as orange, yellow, and green.
(TIF)

S4 Fig Timing of reservoir formation for Participant CAP206. Approximately Maximum-Likelihood trees were used for each of the gene regions; OGV sequences are shown in magenta and proviral sequences are shown in black (non-hypermutated viral DNA) and gray (hypermutated viral DNA). Sequences generated from plasma collected within the first year of diagnosis are shown in shades of red, within the last year before therapy initiation are shown in shades of blue, with times between the first and last year shown as orange, yellow, and green.
(TIF)

S5 Fig Timing of reservoir formation for Participant CAP217. Approximately Maximum-Likelihood trees were used for each of the gene regions; OGV sequences are shown in magenta and proviral sequences are shown in black (non-hypermutated viral DNA) and gray (hypermutated viral DNA). Sequences generated from plasma collected within the first year of diagnosis are shown in shades of red, within the last year before therapy initiation are shown in shades of blue, with times between the first and last year shown as orange, yellow, and green.
(TIF)

S6 Fig Timing of reservoir formation for Participant CAP222. Approximately Maximum-Likelihood trees were used for each of the gene regions; OGV sequences are shown in magenta and proviral sequences are shown in black (non-hypermutated viral DNA) and gray (hypermutated viral DNA). Sequences generated from plasma collected within the first year of diagnosis are shown in shades of red, within the last year before therapy initiation are shown in shades of blue, with times between the first and last year shown as orange, yellow, and green.
(TIF)

S7 Fig Timing of reservoir formation for Participant CAP244. Approximately Maximum-Likelihood trees were used for each of the gene regions. OGV sequences are shown in magenta and proviral sequences are shown in black (non-hypermutated viral DNA) and gray (hypermutated viral DNA). Sequences generated from plasma collected within the first year of diagnosis are shown in shades of red, within the last year before therapy initiation are shown in shades of blue, with times between the first and last year shown as orange, yellow, and green.
(TIF)

S8 Fig Timing of reservoir formation for Participant CAP257. Approximately Maximum-Likelihood trees were used for each of the gene regions. OGV sequences are shown in magenta and proviral sequences are shown in black (non-hypermutated viral DNA) and gray (hypermutated viral DNA). Sequences generated from plasma collected within the first year of diagnosis are shown in shades of red, within the last year before therapy initiation are shown in shades of blue, with times between the first and last year shown as orange, yellow, and green.
(TIF)

S9 Fig Timing of reservoir formation for Participant CAP268. Approximately Maximum-Likelihood trees were used for each of the gene regions. OGV sequences are shown in magenta and proviral sequences are shown in black (non-hypermutated viral DNA) and gray (hypermutated viral DNA). Sequences generated from plasma collected within the first year of diagnosis are shown in shades of red, within the last year before therapy initiation are shown in shades of blue, with times between the first and last year shown as orange, yellow, and green.
(TIF)

S10 Fig Timing of reservoir formation for Participant CAP277. Approximately Maximum-Likelihood trees were used for each of the gene regions. OGV sequences are shown in magenta and proviral sequences are shown in black (non-hypermutated viral DNA) and gray (hypermutated viral DNA). Sequences generated from plasma collected within the first year of diagnosis are shown in shades of red, within the last year before therapy initiation are shown in shades of blue, with times between the first and last year shown as orange, yellow, and green.
(TIF)

S11 Fig Timing of reservoir formation for Participant CAP280. Approximately Maximum-Likelihood trees were used for each of the gene regions. OGV sequences are shown in magenta and proviral sequences are shown in black (non-hypermutated viral DNA) and gray (hypermutated viral DNA). Sequences generated from plasma collected within the first year of diagnosis are shown in shades of red, within the last year before therapy initiation are shown in shades of blue, with times between the first and last year shown as orange, yellow, and green.
(TIF)

S12 Fig Timing of reservoir formation for Participant CAP287. Approximately Maximum-Likelihood trees were used for each of the gene regions. OGV sequences are shown in magenta and proviral sequences are shown in black (non-hypermutated viral DNA) and gray (hypermutated viral DNA). Sequences generated from plasma collected within the first year of diagnosis are shown in shades of red, within the last year before therapy initiation are shown in shades of blue, with times between the first and last year shown as orange, yellow, and green.
(TIF)

S13 Fig Timing of reservoir formation for Participant CAP288. Approximately Maximum-Likelihood trees were used for each of the gene regions. OGV sequences are shown in magenta and proviral sequences are shown in black (non-hypermutated viral DNA) and gray (hypermutated viral DNA). Sequences generated from plasma collected within the first year of diagnosis are shown in shades of red, within the last year before therapy initiation are shown in shades of blue, with times between the first and last year shown as orange, yellow, and green.
(TIF)

S14 Fig Timing of reservoir formation for Participant CAP302. Approximately Maximum-Likelihood trees were used for each of the gene regions. OGV sequences are shown in magenta and proviral sequences are shown in black (non-hypermutated viral DNA) and gray (hypermutated viral DNA). Sequences generated from plasma collected within the first year of diagnosis are shown in shades of red, within the last year before therapy initiation are shown in shades of blue, with times between the first and last year shown as orange, yellow, and green.
(TIF)

S15 Fig Timing of reservoir formation for Participant CAP316. Approximately Maximum-Likelihood trees were used for each of the gene regions. OGV sequences are shown in magenta and proviral sequences are shown in black (non-hypermutated viral DNA) and gray (hypermutated viral DNA). Sequences generated from plasma collected within the first year of diagnosis are shown in shades of red, within the last year before therapy initiation are shown in shades of blue, with times between the first and last year shown as orange, yellow, and green.
(TIF)

S16 Fig Timing of reservoir formation for Participant CAP333. Approximately Maximum-Likelihood trees were used for each of the gene regions. OGV sequences are shown in magenta and proviral sequences are shown in black (non-hypermutated viral DNA) and gray (hypermutated viral DNA). Sequences generated from plasma collected within the first year of diagnosis are shown in shades of red, within the last year before therapy initiation are shown in shades of blue, with times between the first and last year shown as orange, yellow, and green.
(TIF)

S17 Fig Timing of reservoir formation for Participant CAP336. Approximately Maximum-Likelihood trees were used for each of the gene regions. OGV sequences are shown in magenta and proviral sequences are shown in black (non-hypermutated viral DNA) and gray (hypermutated viral DNA). Sequences generated from plasma collected within the first year of diagnosis are shown in shades of red, within the last year before therapy initiation are shown in shades of blue, with times between the first and last year shown as orange, yellow, and green.
(TIF)

S18 Fig Timing of reservoir formation for Participant CAP337. Approximately Maximum-Likelihood trees were used for each of the gene regions. OGV sequences are shown in magenta and proviral sequences are shown in black (non-hypermutated viral DNA) and gray (hypermutated viral DNA). Sequences generated from plasma collected within the first year of diagnosis are shown in shades of red, within the last year before therapy initiation are shown in shades of blue, with times between the first and last year shown as orange, yellow, and green.
(TIF)

S19 Fig Timing of reservoir formation for Participant CAP372. Approximately Maximum-Likelihood trees were used for each of the gene regions. OGV sequences are shown in magenta and proviral sequences are shown in black (non-hypermutated viral DNA) and gray (hypermutated viral DNA). Sequences generated from plasma collected within the first year of diagnosis are shown in shades of red, within the last year before therapy initiation are shown in shades of blue, with times between the first and last year shown as orange, yellow, and green.
(TIF)

S20 Fig Timing of reservoir formation for Participant CAP380. Approximately Maximum-Likelihood trees were used for each of the gene regions. OGV sequences are shown in magenta and proviral sequences are shown in black (non-hypermutated viral DNA) and gray (hypermutated viral DNA). Sequences generated from plasma collected within the first year of diagnosis are shown in shades of red, within the last year before therapy initiation are shown in shades of blue, with times between the first and last year shown as orange, yellow, and green.
(TIF)

S21 Fig Replication-competent and defective viral sequences enter the long-lived reservoir at similar times. Comparisons of the percent of reservoir sequences that entered the reservoir during the year before the initiation of therapy (i.e. the percent late virus) did not detect differences in when different types of variants entered the long-lived reservoir. **A.** Non-hypermutated vDNA and OGVs (Wilcoxon Matched-Pairs Rank Sum; P = 0.91). **B.** Hypermutated vDNA (masked) and non-hypermutated vDNA (Wilcoxon Matched-Pairs Rank Sum; P = 0.14). **C.** OGVs and total vDNA (Wilcoxon Matched-Pairs Rank Sum; P = 0.12). Each line represents one participant and analyses are restricted to participants with at least six sequences of the types being compared.
(TIF)

S22 Fig Masking potentially hypermutated positions does not preclude precise estimation of when variants enter the long-lived reservoir. In order to determine whether masking reduces the dataset too much to accurately date reservoir sequences we examined whether masking APOBEC3G/F recognition sequences precludes accurate dating of control sequences that are not hypermutated (OGVs). Masking did not significantly alter the estimation of the percent of control sequences that form late (Wilcoxon Matched-Pairs Rank Sum; P = 0.68).
(DOCX)
