# Supplementary material for: The timing of HIV-1 infection of cells that persist on therapy is not strongly influenced by replication competency or cellular tropism of the provirus
Source: PLoS Pathog. 2024 Feb 29;20(2):e1011974. doi: 10.1371/journal.ppat.1011974 (PMC10931466; doi:10.1371/journal.ppat.1011974)
Supplement: S1 Table — (DOCX) [file ppat.1011974.s001.docx]

**S1 Table: Masking potential APOBEC sites.**

| Participant | Region Length | | | | # of Masked Sites | | | | # of Potential APOBEC Sites | | | | % of all potential APOBEC sites masked | | | | % of sequence masked | | | |
| --- | --- | --- | --- | --- | --- | --- | --- | --- | --- | --- | --- | --- | --- | --- | --- | --- | --- | --- | --- | --- |
|  | ENV2 | ENV3 | ENV4 | NEF | ENV2 | ENV3 | ENV4 | NEF | ENV2 | ENV3 | ENV4 | NEF | ENV2 | ENV3 | ENV4 | NEF | ENV2 | ENV3 | ENV4 | NEF |
| CAP188_DNA | 282 | 501 | 342 | 465 | 4 | 17 | 9 | 20 | 18 | 48 | 29 | 68 | 24.7 | 34.6 | 31.5 | 29.9 | 1.6 | 3.3 | 2.6 | 4.3 |
| CAP206_DNA | 286 | N/A | 237 | N/A | 4 | N/A | 9 | N/A | 18 | N/A | 28 | N/A | 23.8 | N/A | 33.1 | N/A | 1.5 | N/A | 3.9 | N/A |
| CAP222_DNA | 297 | 501 | 309 | N/A | 1 | 3 | 1 | N/A | 25 | 55 | 34 | N/A | 4.0 | 5.5 | 2.9 | N/A | 0.3 | 0.6 | 0.3 | N/A |
| CAP244_DNA | 279 | 497 | 381 | 433 | 6 | 13 | 11 | 14 | 22 | 49 | 35 | 64 | 26.5 | 27.4 | 30.6 | 22.5 | 2.1 | 2.7 | 2.8 | 3.3 |
| CAP257_DNA | 286 | 478 | 226 | 412 | 3 | 13 | 9 | 19 | 19 | 52 | 33 | 67 | 15.7 | 25.2 | 28.6 | 28.7 | 1.0 | 2.8 | 4.2 | 4.6 |
| CAP268_DNA | 369 | 484 | 305 | 418 | 5 | 12 | 7 | 14 | 30 | 50 | 32 | 65 | 16.8 | 24.2 | 23.1 | 21.2 | 1.4 | 2.5 | 2.4 | 3.3 |
| CAP277_DNA | 288 | N/A | 336 | 426 | 3 | N/A | 8 | 16 | 17 | N/A | 33 | 71 | 14.4 | N/A | 24.9 | 21.9 | 0.9 | N/A | 2.5 | 3.7 |
| CAP280_DNA | 283 | 481 | 303 | 432 | 5 | 12 | 14 | 17 | 25 | 52 | 44 | 75 | 19.2 | 22.9 | 30.5 | 21.9 | 1.7 | 2.5 | 4.5 | 3.8 |
| CAP287_DNA | 291 | 433 | 272 | N/A | 7 | 14 | 12 | N/A | 22 | 50 | 40 | N/A | 30.6 | 28.8 | 29.4 | N/A | 2.3 | 3.3 | 4.3 | N/A |
| CAP302_DNA | N/A | 472 | 232 | 417 | N/A | 9 | 9 | 16 | N/A | 49 | 38 | 70 | N/A | 18.2 | 24.8 | 23.1 | N/A | 1.9 | 4.1 | 3.9 |
| CAP316_DNA | 277 | 478 | 232 | N/A | 13 | 34 | 20 | N/A | 18 | 49 | 28 | N/A | 70.2 | 70.5 | 69.1 | N/A | 4.5 | 7.2 | 8.4 | N/A |
| CAP333_DNA | N/A | 486 | 282 | 423 | N/A | 17 | 14 | 23 | N/A | 43 | 34 | 73 | N/A | 40.1 | 40.6 | 31.9 | N/A | 3.6 | 4.8 | 5.5 |
| CAP336_DNA | 322 | 478 | 289 | 420 | 7 | 14 | 14 | 18 | 28 | 45 | 41 | 68 | 26.8 | 30.8 | 34.0 | 26.4 | 2.3 | 2.9 | 4.8 | 4.3 |
| CAP337_DNA | 274 | 478 | 220 | 420 | 7 | 19 | 13 | 21 | 19 | 46 | 29 | 68 | 38.1 | 40.6 | 45.7 | 31.4 | 2.7 | 3.9 | 6.1 | 5.1 |
| CAP372_DNA | 286 | 478 | 248 | 423 | 4 | 13 | 11 | 19 | 21 | 48 | 36 | 70 | 19.9 | 25.9 | 30.7 | 27.5 | 1.5 | 2.6 | 4.4 | 4.5 |
| CAP380_DNA | 292 | 478 | 234 | 439 | 5 | 20 | 10 | 22 | 25 | 56 | 38 | 72 | 20.0 | 35.2 | 25.1 | 29.9 | 1.7 | 4.1 | 4.1 | 4.9 |
| **AVERAGE** | **293.7** | **480.2** | **278.0** | **427.3** | **5.3** | **15.0** | **10.7** | **18.2** | **22.0** | **49.4** | **34.5** | **69.2** | **25.1** | **30.7** | **31.5** | **26.4** | **1.8** | **3.1** | **4.0** | **4.3** |
