# Supplementary material for: The timing of HIV-1 infection of cells that persist on therapy is not strongly influenced by replication competency or cellular tropism of the provirus
Source: PLoS Pathog. 2024 Feb 29;20(2):e1011974. doi: 10.1371/journal.ppat.1011974 (PMC10931466; doi:10.1371/journal.ppat.1011974)
Supplement: S2 Table — (DOCX) [file ppat.1011974.s002.docx]

**S2 Table:** Analysis to determine if OGVs were preferentially eliminated over time from the pool of long-lived cells.

| **Participant** | **Timing of reservoir formation** | **Number of reservoir sequences** | | **Odds ratio** | **95% confidence interval** | | **p-value** | **bonferroni corrected p-value** |
| --- | --- | --- | --- | --- | --- | --- | --- | --- |
|  |  | **OGV** | **DNA** |  |  |  |  |  |
| CAP188 | Early | 4 | 15 | 0.45 | 0.08 | 2.08 | 0.32 | 1.00 |
|  | Late | 9 | 15 |  |  |  |  |  |
| CAP206 | Early | 8 | 7 | 0.73 | 0.15 | 3.60 | 0.73 | 1.00 |
|  | Late | 11 | 7 |  |  |  |  |  |
| CAP217 | Early | 8 | 8 | 1.00 | 0.20 | 4.93 | 1.00 | 1.00 |
|  | Late | 8 | 8 |  |  |  |  |  |
| CAP257 | Early | 3 | 7 | 0.25 | 0.04 | 1.20 | 0.08 | 0.79 |
|  | Late | 41 | 23 |  |  |  |  |  |
| CAP287 | Early | 2 | 14 | 0.58 | 0.05 | 4.79 | 0.67 | 1.00 |
|  | Late | 4 | 16 |  |  |  |  |  |
| CAP288 | Early | 0 | 11 | 0.00 | 0.00 | 3.56 | 0.59 | 1.00 |
|  | Late | 7 | 48 |  |  |  |  |  |
| CAP302 | Early | 5 | 32 | 2.16 | 0.21 | 110.68 | 0.66 | 1.00 |
|  | Late | 1 | 14 |  |  |  |  |  |
| CAP316 | Early | 1 | 7 | 0.23 | 0.00 | 2.21 | 0.23 | 1.00 |
|  | Late | 12 | 19 |  |  |  |  |  |
| CAP336 | Early | 0 | 11 | 0.00 | 0.00 | 1.00 | 0.04 | 0.41 |
|  | Late | 11 | 22 |  |  |  |  |  |
| CAP372 | Early | 5 | 24 | 1.45 | 0.30 | 7.06 | 0.73 | 1.00 |
|  | Late | 5 | 35 |  |  |  |  |  |
