# Supplementary material for: The timing of HIV-1 infection of cells that persist on therapy is not strongly influenced by replication competency or cellular tropism of the provirus
Source: PLoS Pathog. 2024 Feb 29;20(2):e1011974. doi: 10.1371/journal.ppat.1011974 (PMC10931466; doi:10.1371/journal.ppat.1011974)
Supplement: S3 Table — (DOCX) [file ppat.1011974.s003.docx]

**S3 Table: Primer Sequences.**

| **Amplicon Name** | **Forward Sequence** | **Reverse Sequence** | **Overlapping** |
| --- | --- | --- | --- |
| GAG1 | NNNNATGGGTGCGAGAGCGTCARTATTA | NNNNNNNNNNNACATGGGTATTACCTCTGGGCT | yes |
| GAG2 | NNNNCACCTAGAACYTTGAATGCATG | NNNNNNNNNNNTCTAATGTAGCCCCTGGTCCTAAT | no |
| POL1 | NNNNAGCAGAGAGCTTCAGGTTCG | NNNNNNNNNNNTCTTCTGTCAATGGCCATTGT | yes |
| POL1 | NNNNCTGTACCAGTAAAAYTRAAGCCAGGAATG | NNNNNNNNNNNCTACTCTGGAATATTGCTGGTGATCC | yes |
| POL2 | NNNNCTGCATTCACCATACCTAGTATAAAC | NNNNNNNNNNNTGATGGGTCATAATATACTCCATG | no |
| POL3 | NNNNGGCTACATAGAAGCAGAGGT | NNNNNNNNNNNAATCTTCATCCTGTCTACCTGCCACAC | no |
| POL4 | NNNNGTGACATAAAGGTAGTACCAAGGAGGA | NNNNNNNNNNNGCTGTCAGTGCCAAGTATTGTAGA | yes |
| VIF | NNNNGCCATAAGAAAAGCCATATTAGGAC | NNNNNNNNNNNCTTTTGTCTGAAAGCAAACTAGACAATG | yes |
| ENV1 | NNNNGAGCAGAAGACAGTGGCAATGAGAGT | NNNNNNNNNNNAGGCTTTGATCCCATAAACTGATTATATC | yes |
| ENV2 | NNNNATGGGATCAAAGCCTAAARCCATGTGTA | NNNNNNNNNNNCTTAATTCCATGTGTACATTGTACTGTRCT | yes |
| ENV3 | NNNNACCTCAGCCATAACACAAGCCTG | NNNNNNNNNNNGCTATGTGTTGTAATTTCTAGRTCCCCT | yes |
| ENV4 | NNNNACACATAGCTTTAATTGTRGAGGAGAATTT | NNNNNNNNNNNTGCTATTCCCAATGGCTTAATTTCTACYAC | yes |
| ENV5 | NNNNGGCTGTGGTATATAAAAATATTCATMATGA | NNNNNNNNNNNCTTATTCTTCTAGGTATGTTGHRG | yes |
| NEF | NNNNATAAGACAGGGCTTTGAAGCAGC | NNNNNNNNNNNAGCACCATCCAAAGGTCAGTGG | yes |
