# Supplementary figures and images for: The timing of HIV-1 infection of cells that persist on therapy is not strongly influenced by replication competency or cellular tropism of the provirus
Source: PLoS Pathog. 2024 Feb 29;20(2):e1011974. doi: 10.1371/journal.ppat.1011974 (PMC10931466; doi:10.1371/journal.ppat.1011974)

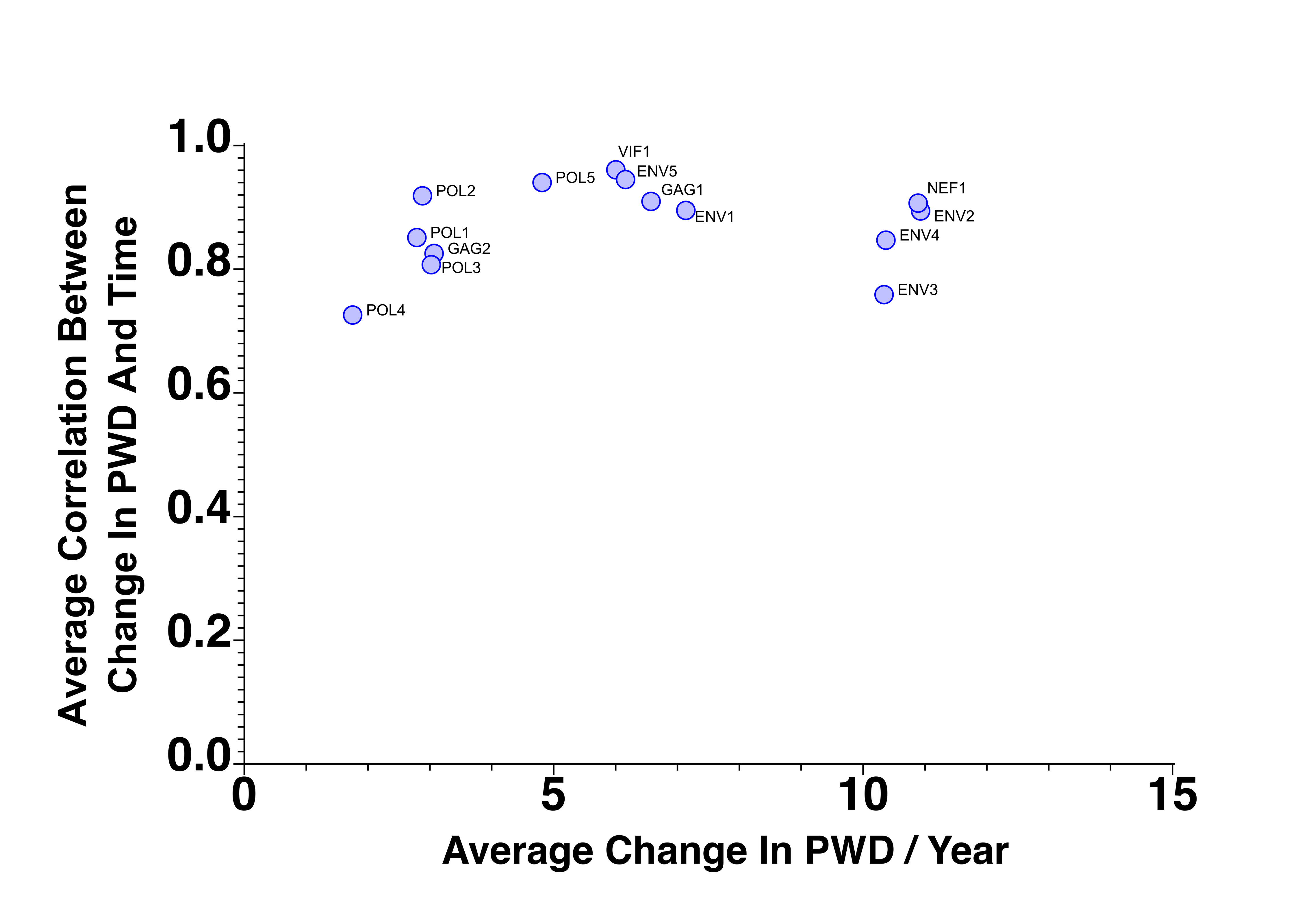

Supplement: S1 Fig — Thirteen amplicons distributed across the HIV-1 genome were used to analyze RNA genomes isolated from the plasma of 18 participants at an average of 10 pre-ART timepoints. For each amplicon the average pairwise distance (PWD) was calculated between sequences at that timepoint and a consensus from the first timepoint. This was repeated at each timepoint during untreated infection and the relationship between change in PWD and weeks between timepoints was analyzed. For each amplicon, we show the average increase in pairwise distance per year and the correlation between change in PWD and time. (TIF) [file ppat.1011974.s004.tif]

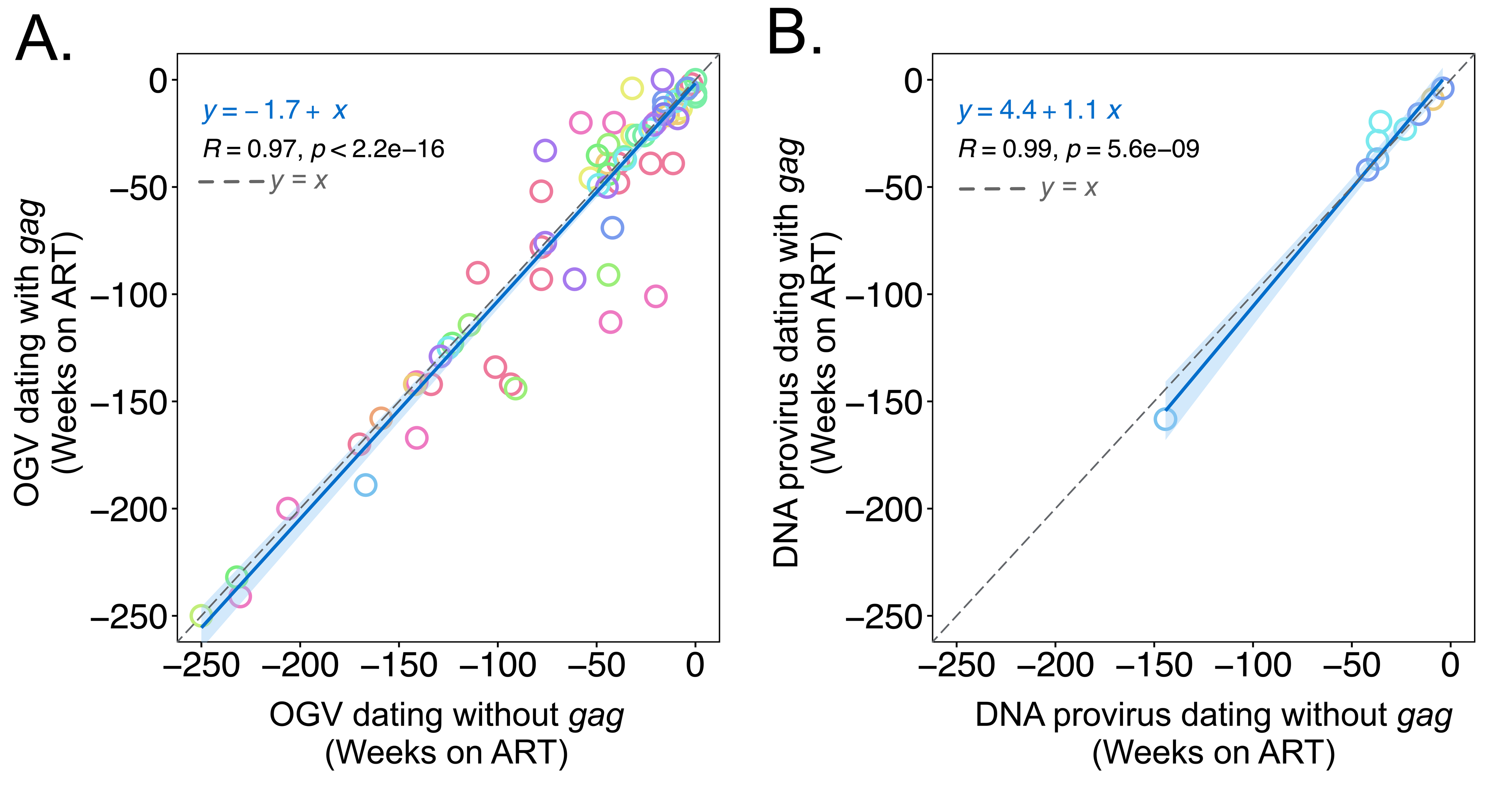

Supplement: S2 Fig — The addition of a gag amplicon in the 5’ half genome did not have a major impact on the estimated date of individual A. OGV or B. proviruses. Each circle represents a sequence in long-lived cells and colors correspond to different participants. (TIF) [file ppat.1011974.s005.tif]

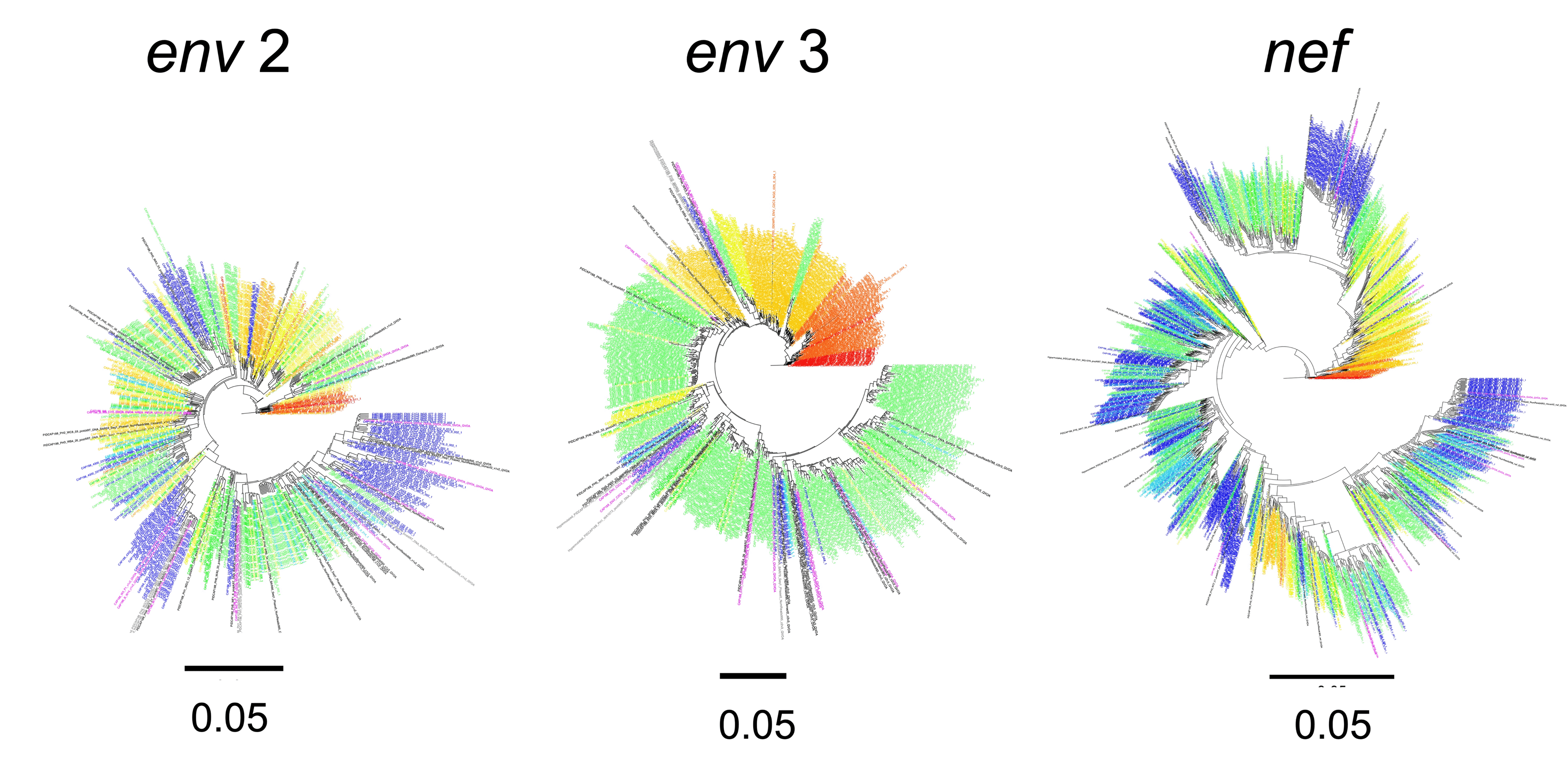

Supplement: S3 Fig — Approximately Maximum-Likelihood trees were used for each of the gene regions; OGV sequences are shown in magenta and proviral sequences are shown in black (non-hypermutated viral DNA) and gray (hypermutated viral DNA). Sequences generated from plasma collected within the first year of diagnosis are shown in shades of red, within the last year before therapy initiation are shown in shades of blue, with times between the first and last year shown as orange, yellow, and green. (TIF) [file ppat.1011974.s006.tif]

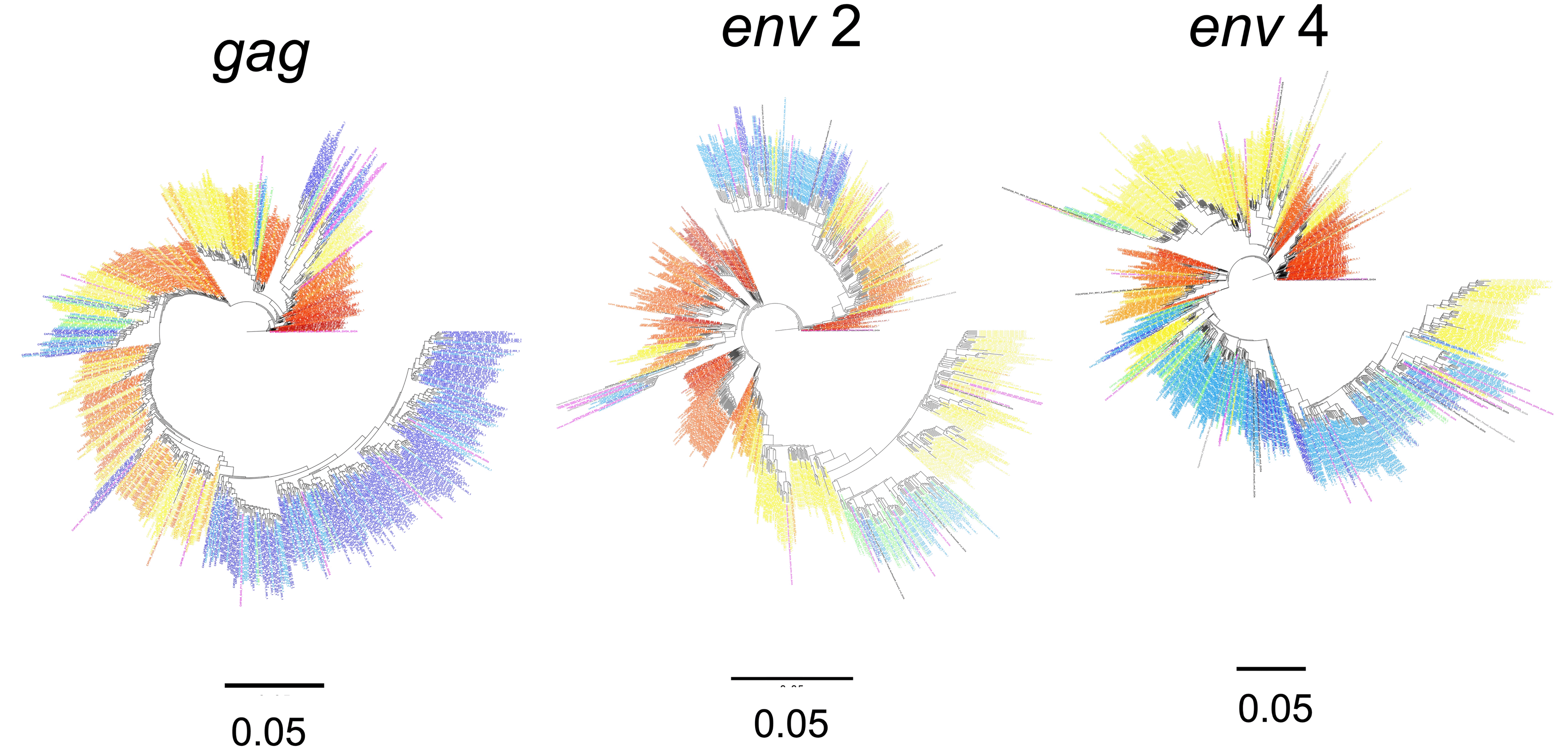

Supplement: S4 Fig — Approximately Maximum-Likelihood trees were used for each of the gene regions; OGV sequences are shown in magenta and proviral sequences are shown in black (non-hypermutated viral DNA) and gray (hypermutated viral DNA). Sequences generated from plasma collected within the first year of diagnosis are shown in shades of red, within the last year before therapy initiation are shown in shades of blue, with times between the first and last year shown as orange, yellow, and green. (TIF) [file ppat.1011974.s007.tif]

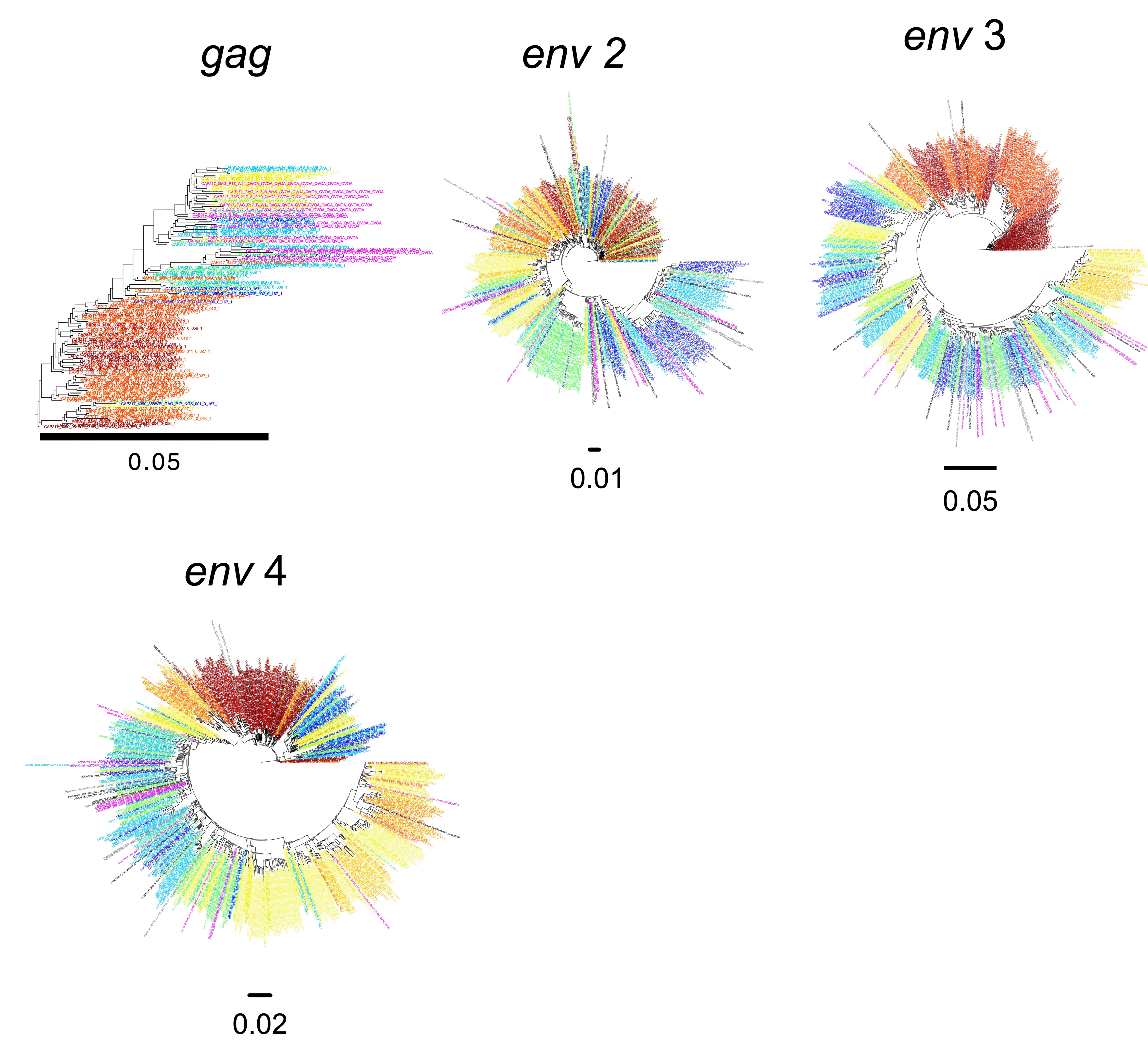

Supplement: S5 Fig — Approximately Maximum-Likelihood trees were used for each of the gene regions; OGV sequences are shown in magenta and proviral sequences are shown in black (non-hypermutated viral DNA) and gray (hypermutated viral DNA). Sequences generated from plasma collected within the first year of diagnosis are shown in shades of red, within the last year before therapy initiation are shown in shades of blue, with times between the first and last year shown as orange, yellow, and green. (TIF) [file ppat.1011974.s008.tif]

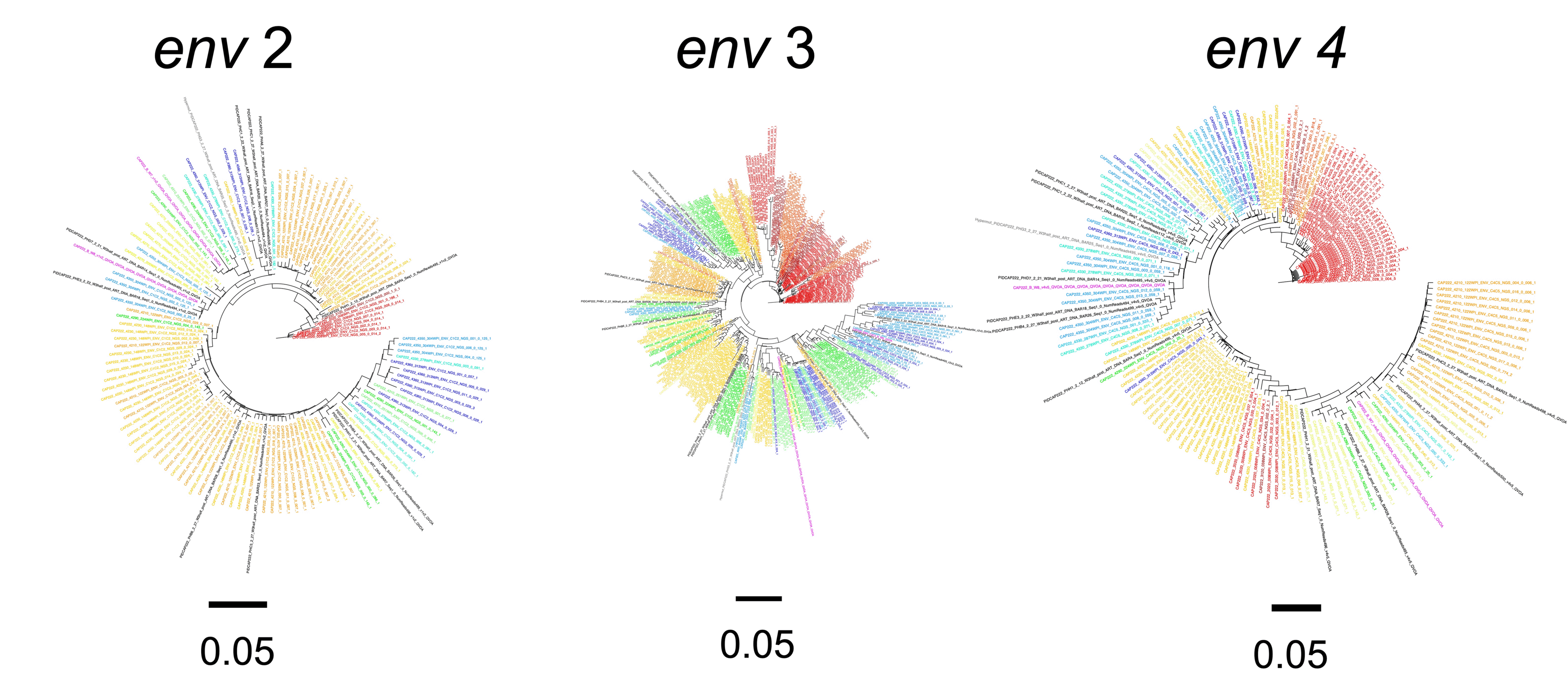

Supplement: S6 Fig — Approximately Maximum-Likelihood trees were used for each of the gene regions; OGV sequences are shown in magenta and proviral sequences are shown in black (non-hypermutated viral DNA) and gray (hypermutated viral DNA). Sequences generated from plasma collected within the first year of diagnosis are shown in shades of red, within the last year before therapy initiation are shown in shades of blue, with times between the first and last year shown as orange, yellow, and green. (TIF) [file ppat.1011974.s009.tif]

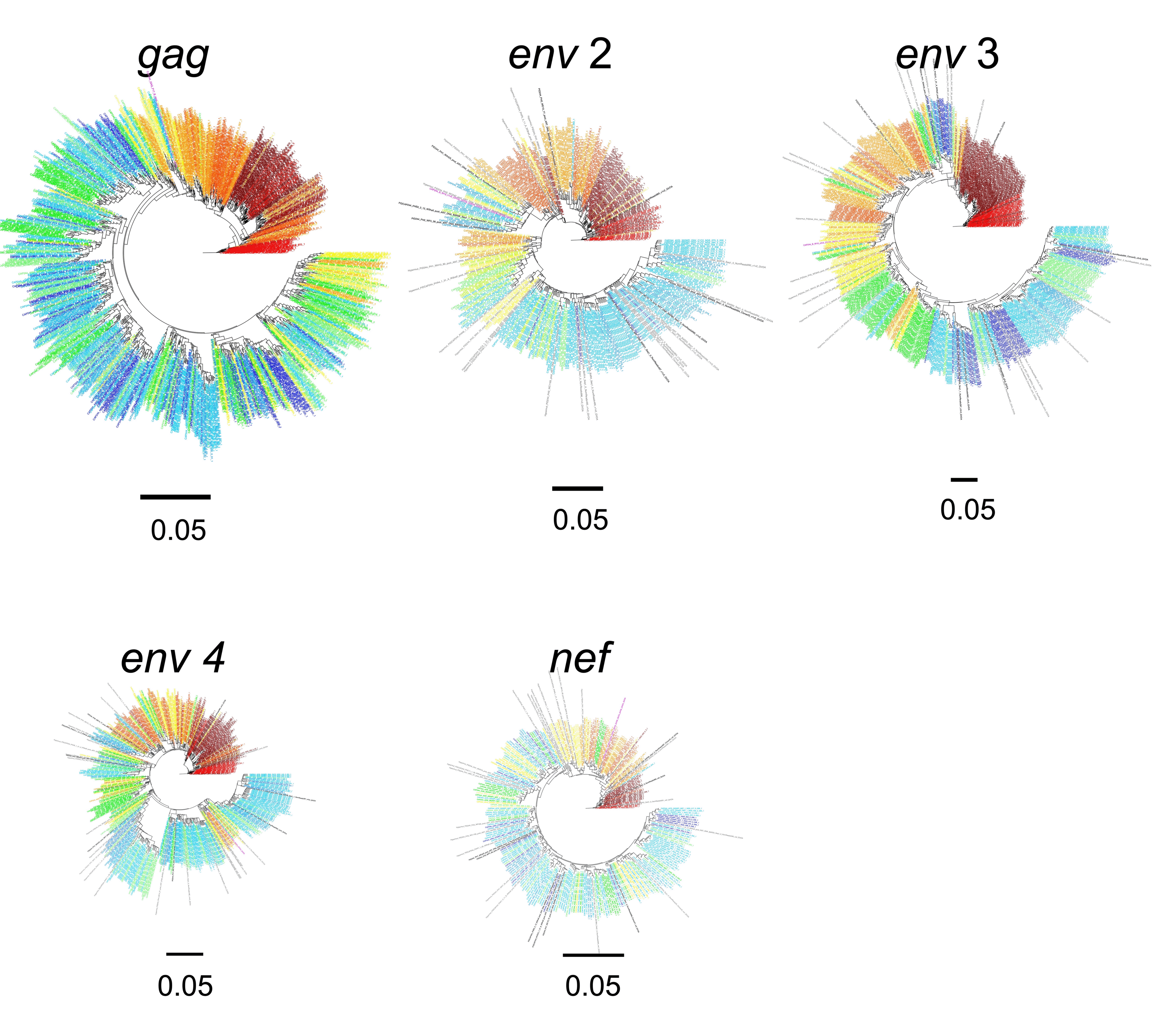

Supplement: S7 Fig — Approximately Maximum-Likelihood trees were used for each of the gene regions. OGV sequences are shown in magenta and proviral sequences are shown in black (non-hypermutated viral DNA) and gray (hypermutated viral DNA). Sequences generated from plasma collected within the first year of diagnosis are shown in shades of red, within the last year before therapy initiation are shown in shades of blue, with times between the first and last year shown as orange, yellow, and green. (TIF) [file ppat.1011974.s010.tif]

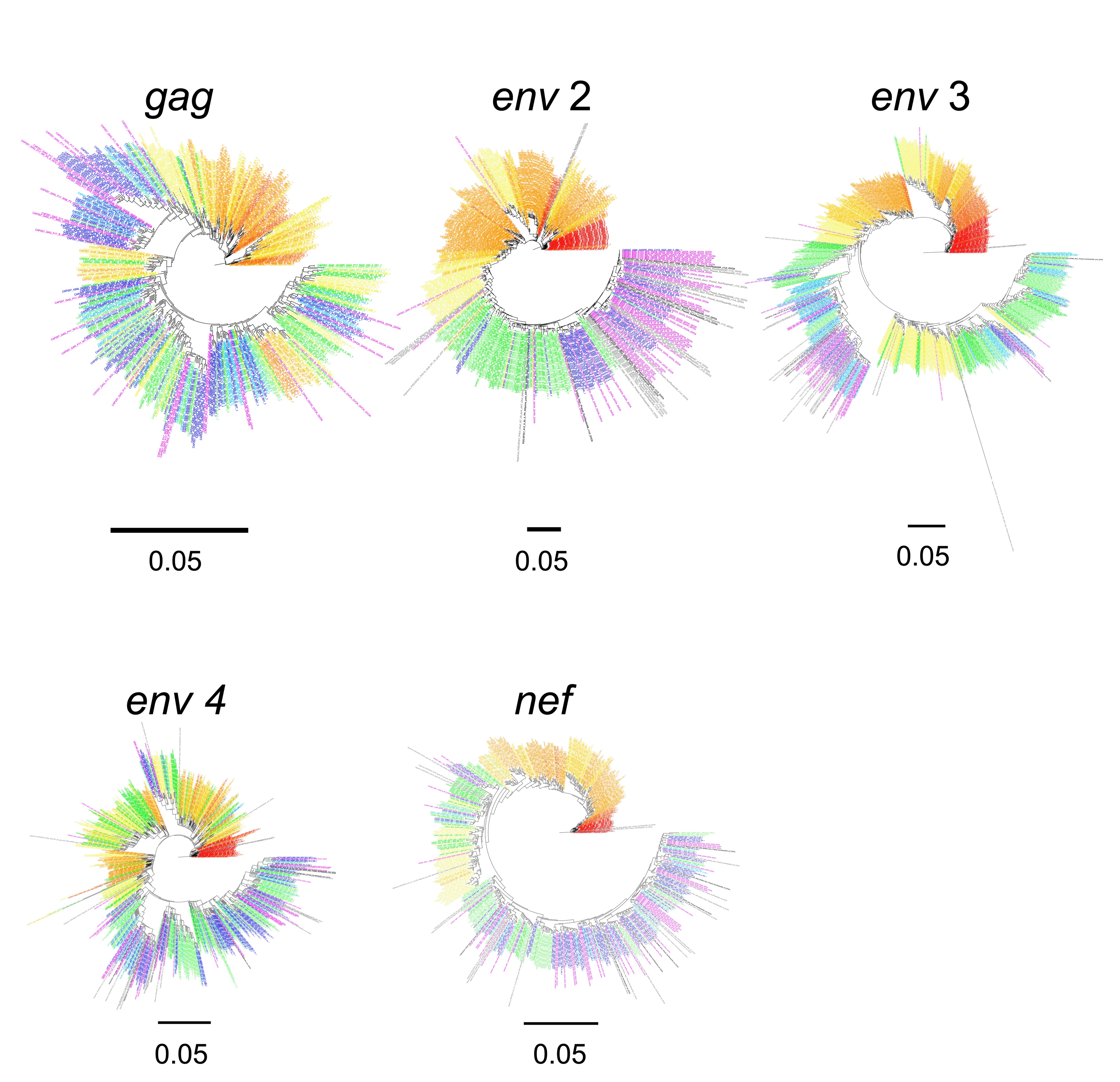

Supplement: S8 Fig — Approximately Maximum-Likelihood trees were used for each of the gene regions. OGV sequences are shown in magenta and proviral sequences are shown in black (non-hypermutated viral DNA) and gray (hypermutated viral DNA). Sequences generated from plasma collected within the first year of diagnosis are shown in shades of red, within the last year before therapy initiation are shown in shades of blue, with times between the first and last year shown as orange, yellow, and green. (TIF) [file ppat.1011974.s011.tif]

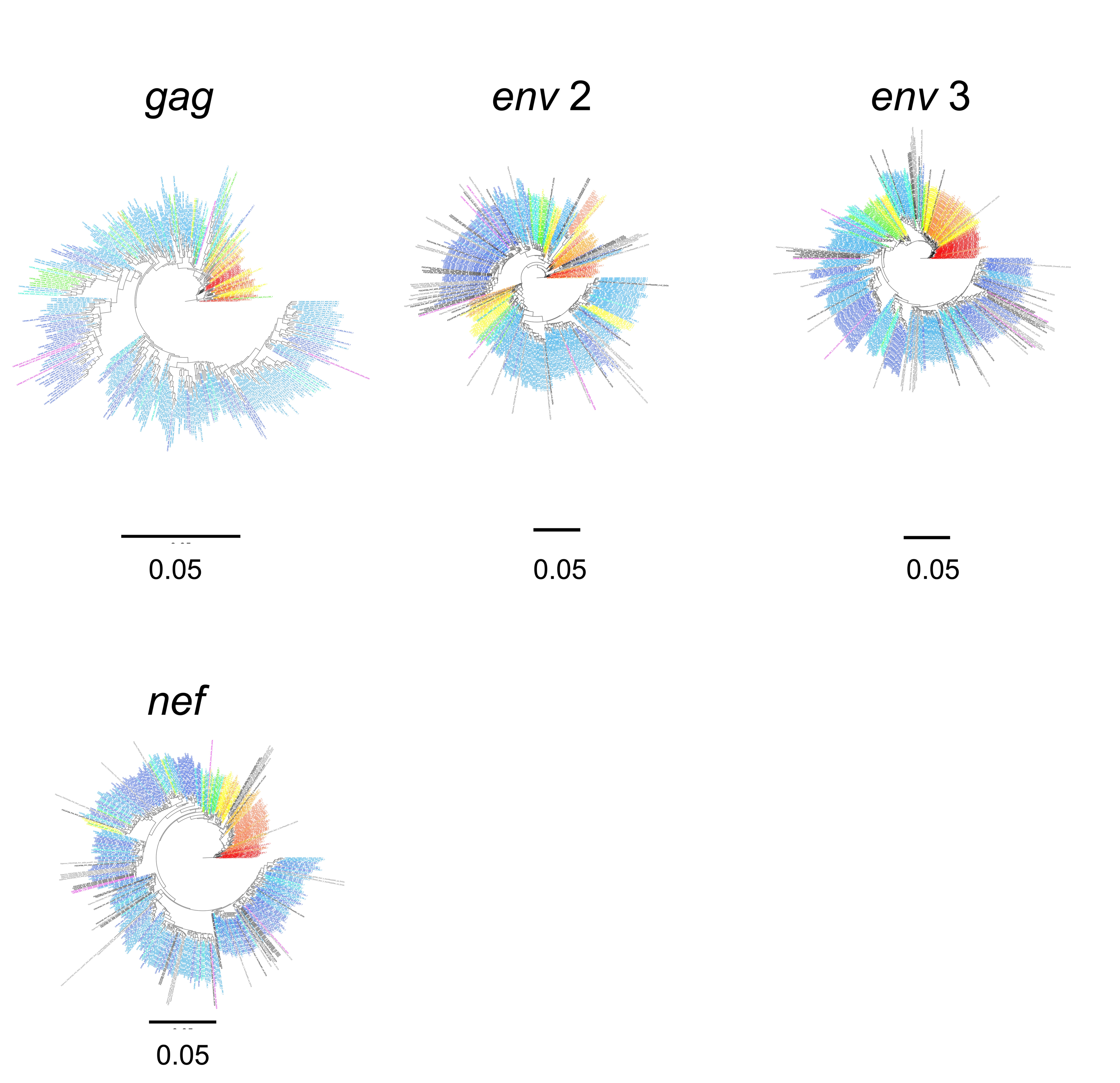

Supplement: S9 Fig — Approximately Maximum-Likelihood trees were used for each of the gene regions. OGV sequences are shown in magenta and proviral sequences are shown in black (non-hypermutated viral DNA) and gray (hypermutated viral DNA). Sequences generated from plasma collected within the first year of diagnosis are shown in shades of red, within the last year before therapy initiation are shown in shades of blue, with times between the first and last year shown as orange, yellow, and green. (TIF) [file ppat.1011974.s012.tif]

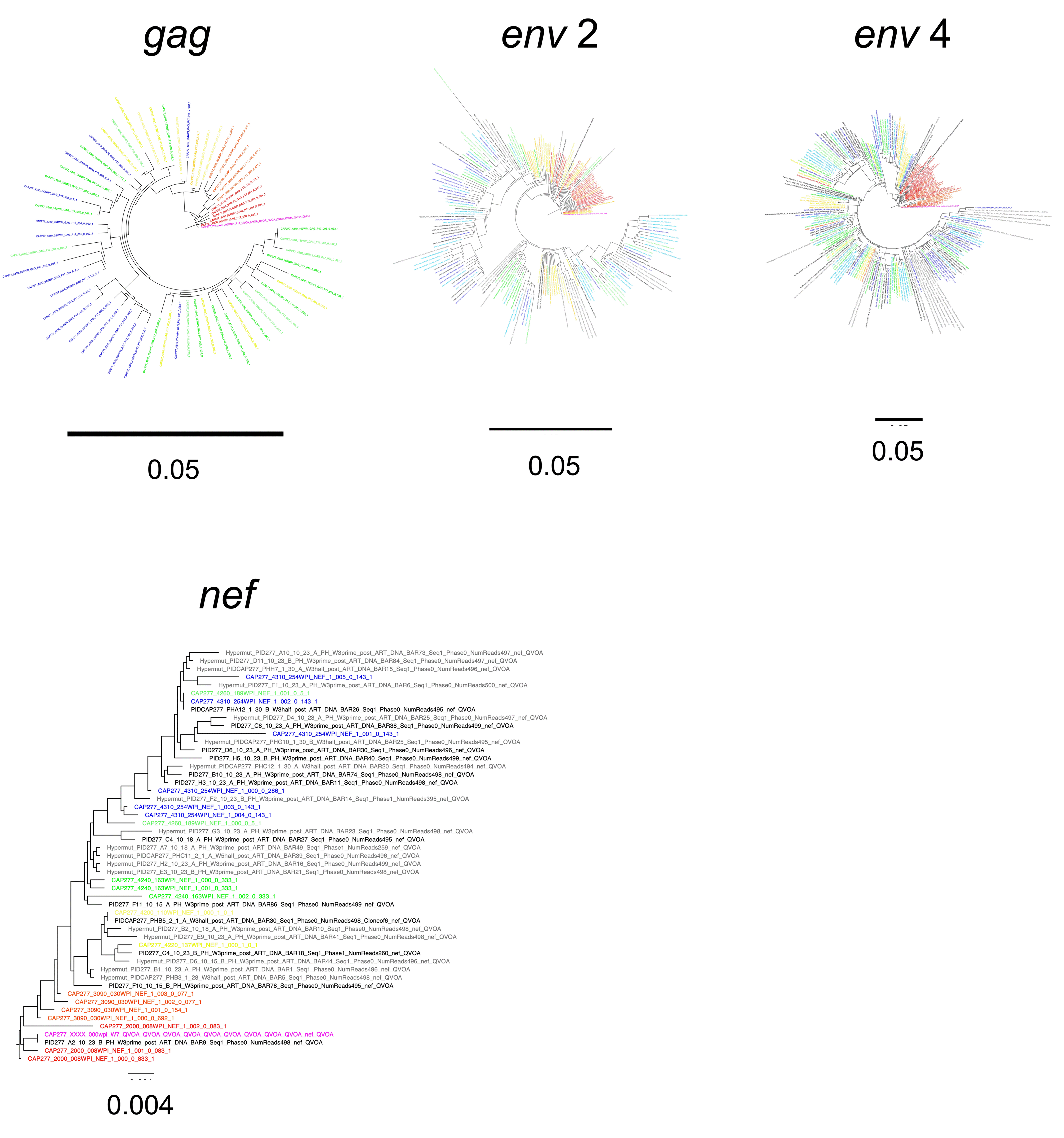

Supplement: S10 Fig — Approximately Maximum-Likelihood trees were used for each of the gene regions. OGV sequences are shown in magenta and proviral sequences are shown in black (non-hypermutated viral DNA) and gray (hypermutated viral DNA). Sequences generated from plasma collected within the first year of diagnosis are shown in shades of red, within the last year before therapy initiation are shown in shades of blue, with times between the first and last year shown as orange, yellow, and green. (TIF) [file ppat.1011974.s013.tif]

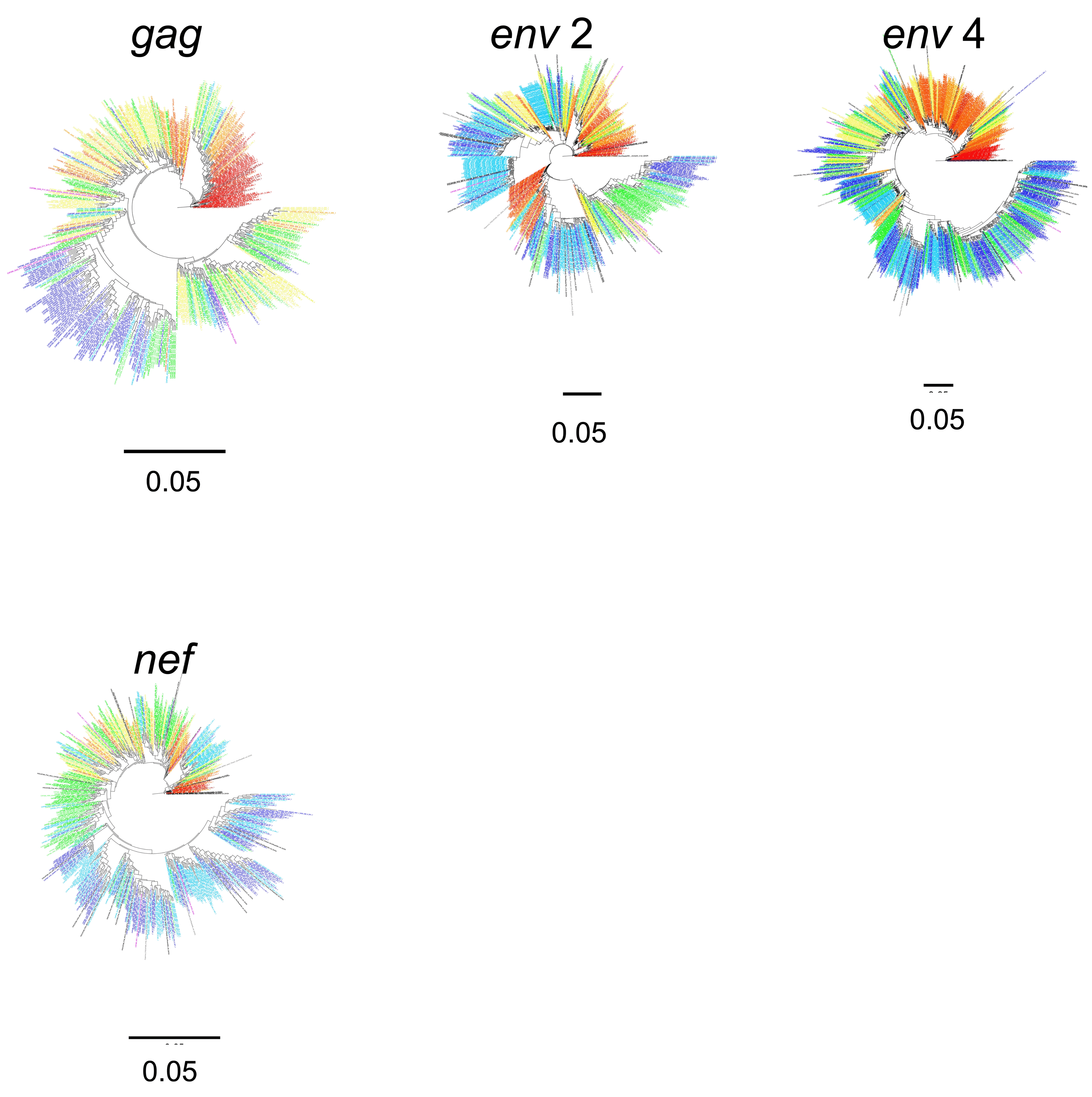

Supplement: S11 Fig — Approximately Maximum-Likelihood trees were used for each of the gene regions. OGV sequences are shown in magenta and proviral sequences are shown in black (non-hypermutated viral DNA) and gray (hypermutated viral DNA). Sequences generated from plasma collected within the first year of diagnosis are shown in shades of red, within the last year before therapy initiation are shown in shades of blue, with times between the first and last year shown as orange, yellow, and green. (TIF) [file ppat.1011974.s014.tif]

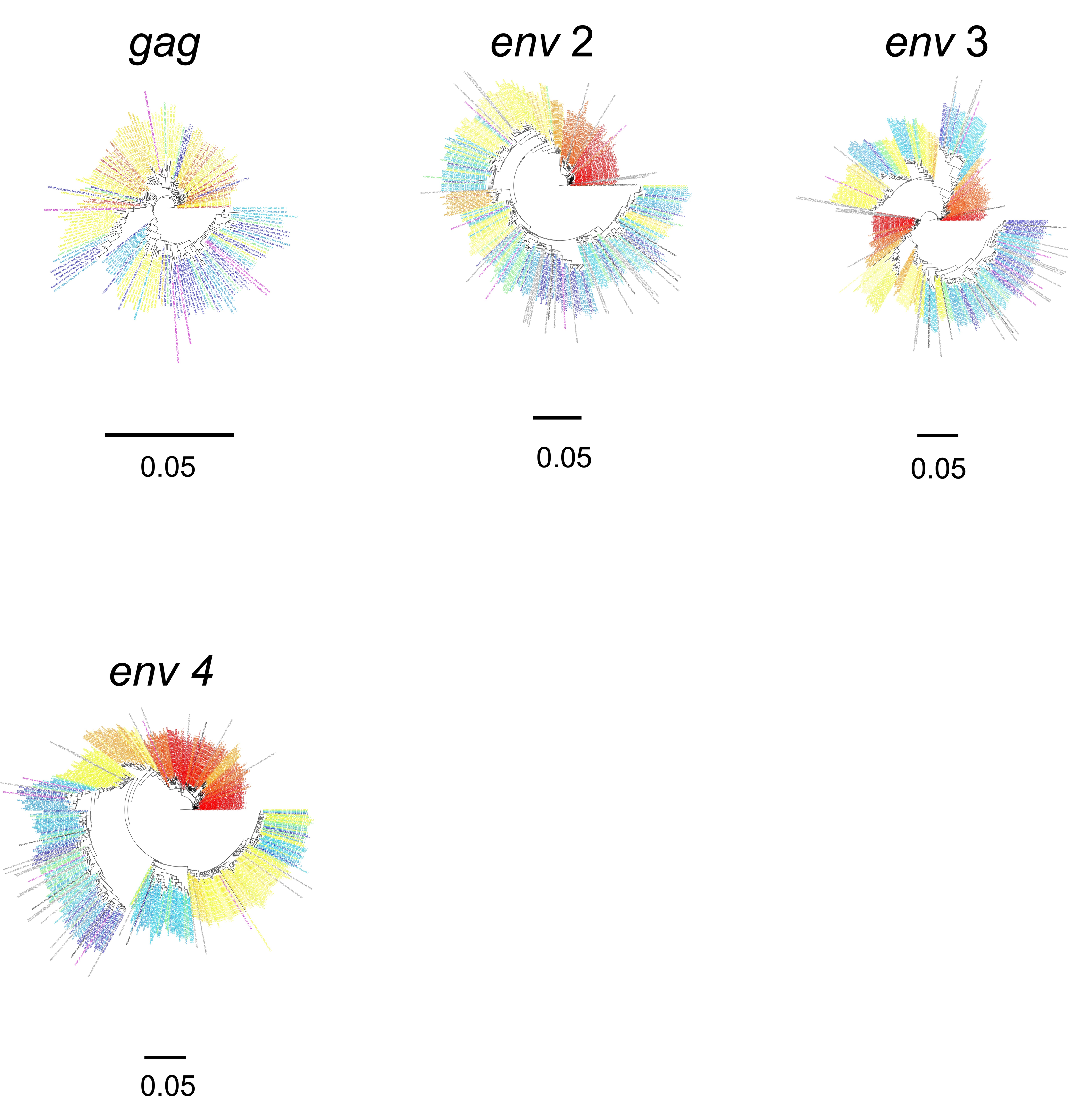

Supplement: S12 Fig — Approximately Maximum-Likelihood trees were used for each of the gene regions. OGV sequences are shown in magenta and proviral sequences are shown in black (non-hypermutated viral DNA) and gray (hypermutated viral DNA). Sequences generated from plasma collected within the first year of diagnosis are shown in shades of red, within the last year before therapy initiation are shown in shades of blue, with times between the first and last year shown as orange, yellow, and green. (TIF) [file ppat.1011974.s015.tif]

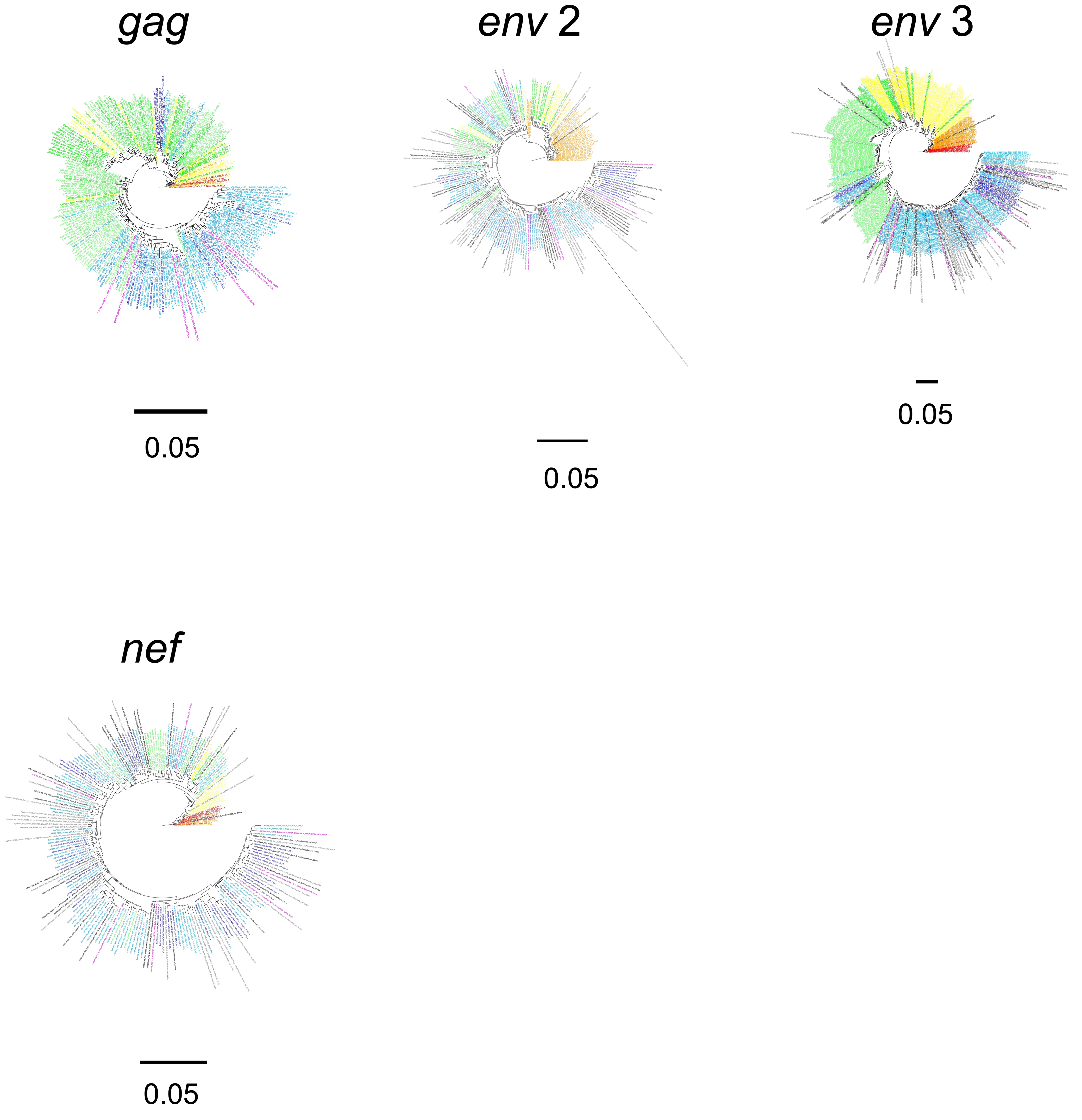

Supplement: S13 Fig — Approximately Maximum-Likelihood trees were used for each of the gene regions. OGV sequences are shown in magenta and proviral sequences are shown in black (non-hypermutated viral DNA) and gray (hypermutated viral DNA). Sequences generated from plasma collected within the first year of diagnosis are shown in shades of red, within the last year before therapy initiation are shown in shades of blue, with times between the first and last year shown as orange, yellow, and green. (TIF) [file ppat.1011974.s016.tif]

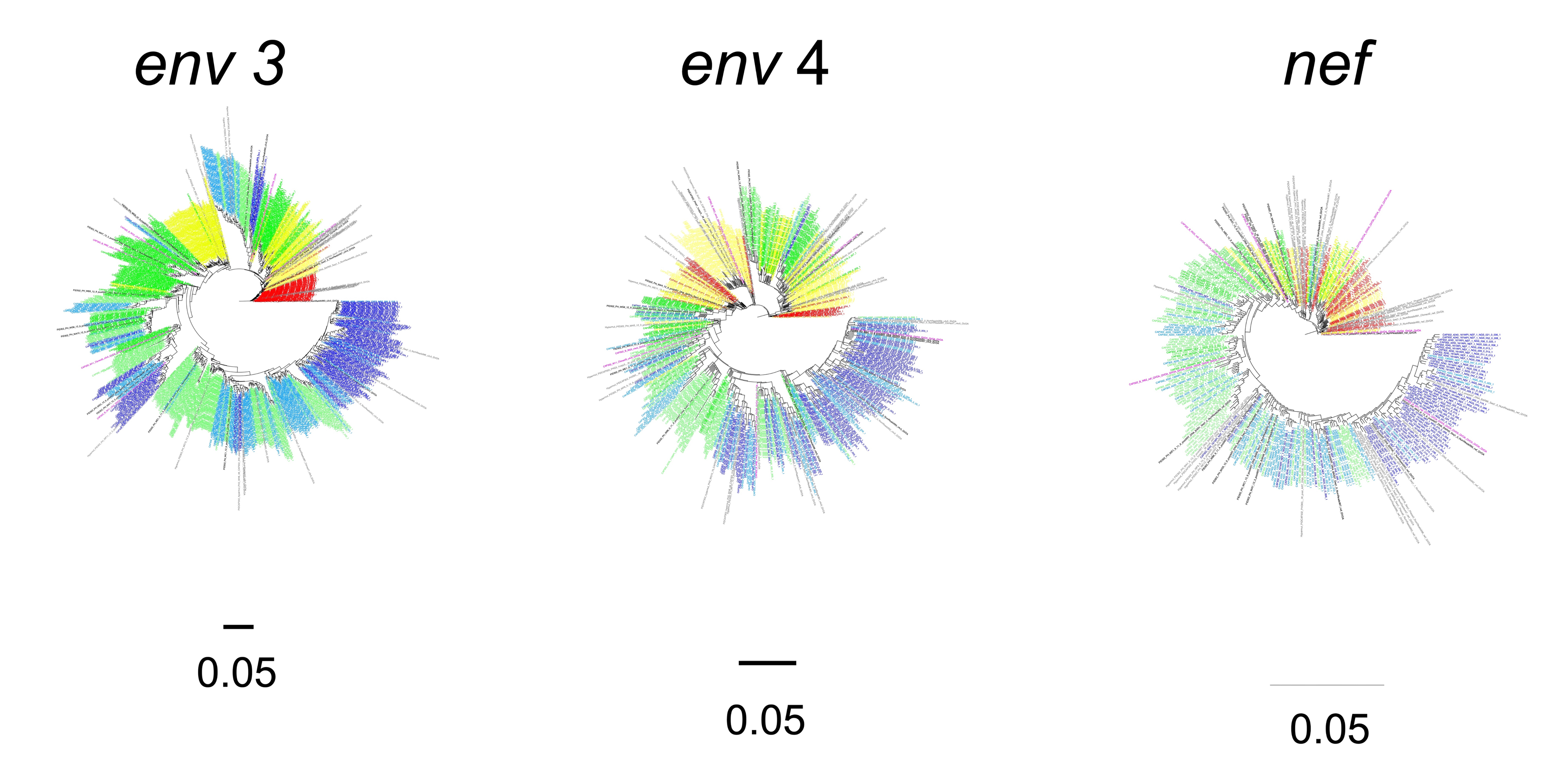

Supplement: S14 Fig — Approximately Maximum-Likelihood trees were used for each of the gene regions. OGV sequences are shown in magenta and proviral sequences are shown in black (non-hypermutated viral DNA) and gray (hypermutated viral DNA). Sequences generated from plasma collected within the first year of diagnosis are shown in shades of red, within the last year before therapy initiation are shown in shades of blue, with times between the first and last year shown as orange, yellow, and green. (TIF) [file ppat.1011974.s017.tif]

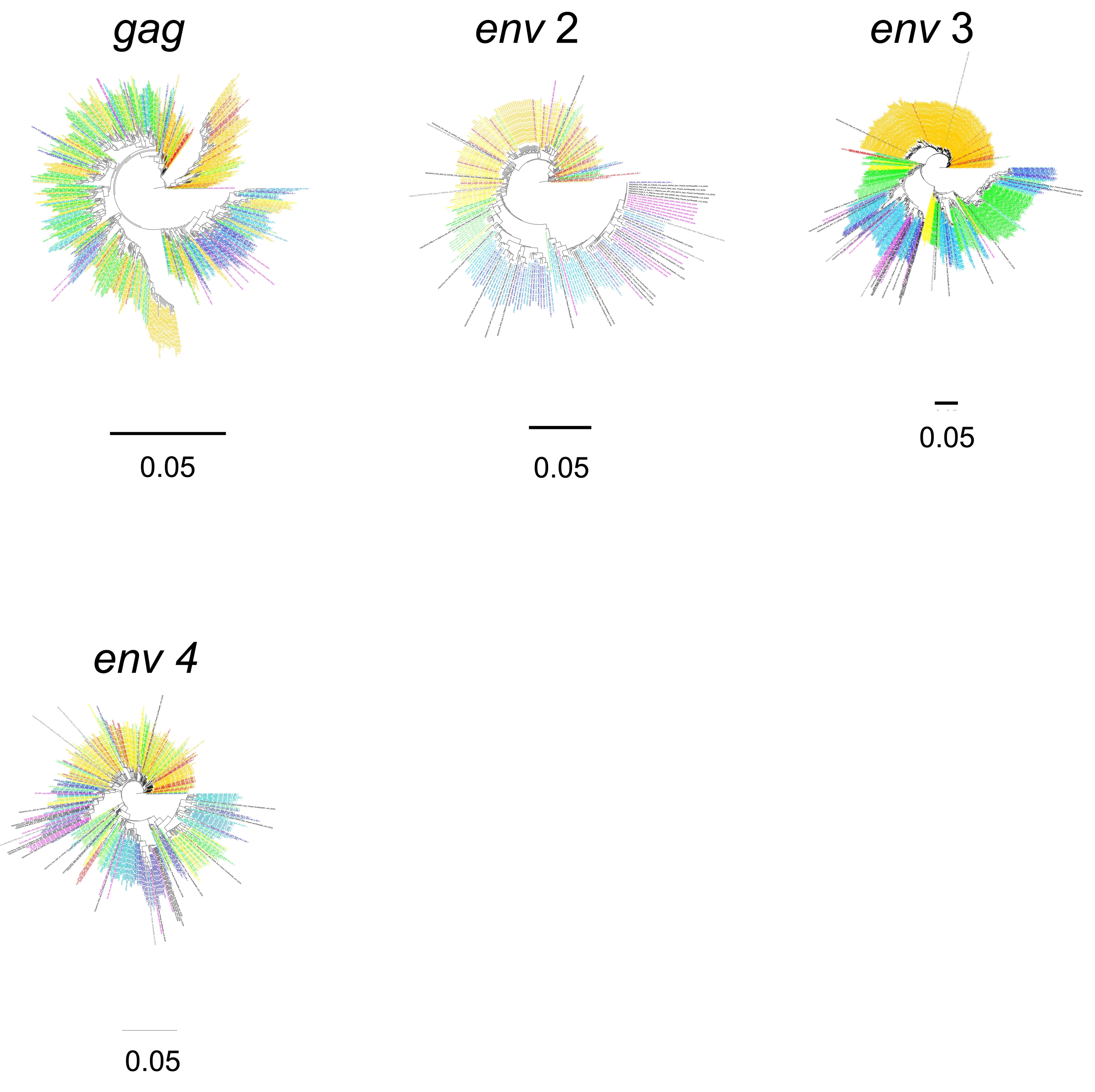

Supplement: S15 Fig — Approximately Maximum-Likelihood trees were used for each of the gene regions. OGV sequences are shown in magenta and proviral sequences are shown in black (non-hypermutated viral DNA) and gray (hypermutated viral DNA). Sequences generated from plasma collected within the first year of diagnosis are shown in shades of red, within the last year before therapy initiation are shown in shades of blue, with times between the first and last year shown as orange, yellow, and green. (TIF) [file ppat.1011974.s018.tif]

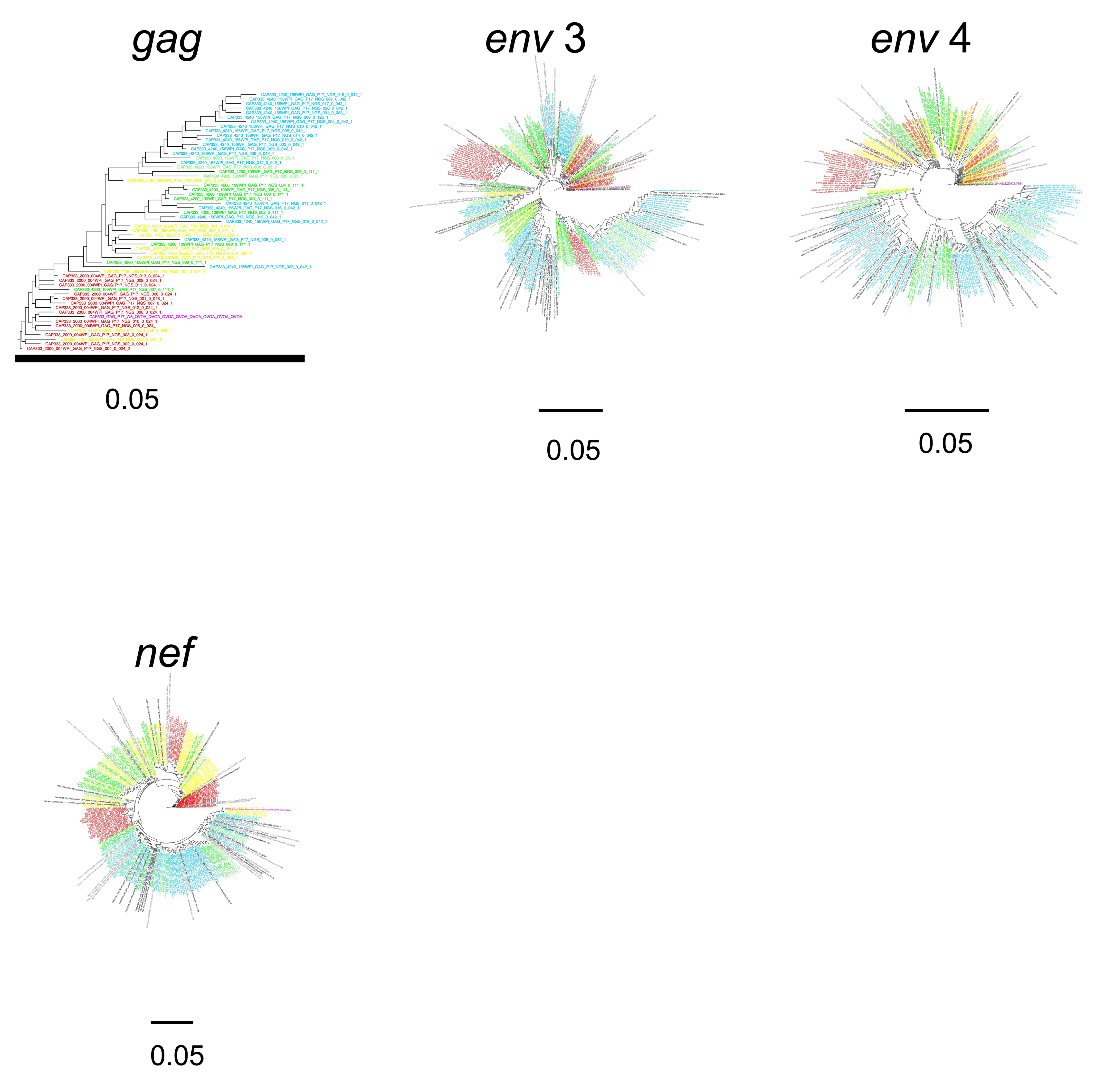

Supplement: S16 Fig — Approximately Maximum-Likelihood trees were used for each of the gene regions. OGV sequences are shown in magenta and proviral sequences are shown in black (non-hypermutated viral DNA) and gray (hypermutated viral DNA). Sequences generated from plasma collected within the first year of diagnosis are shown in shades of red, within the last year before therapy initiation are shown in shades of blue, with times between the first and last year shown as orange, yellow, and green. (TIF) [file ppat.1011974.s019.tif]

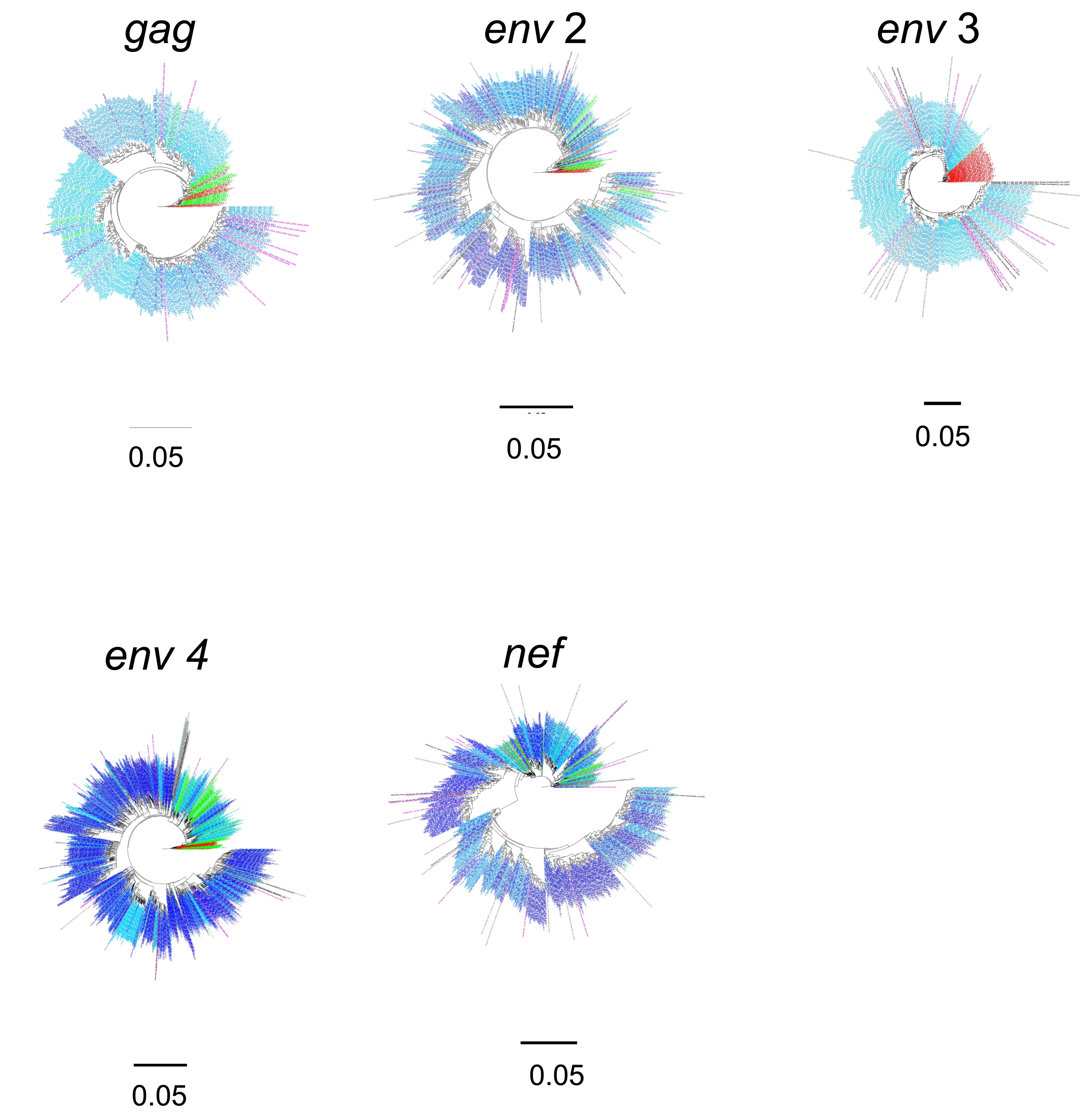

Supplement: S17 Fig — Approximately Maximum-Likelihood trees were used for each of the gene regions. OGV sequences are shown in magenta and proviral sequences are shown in black (non-hypermutated viral DNA) and gray (hypermutated viral DNA). Sequences generated from plasma collected within the first year of diagnosis are shown in shades of red, within the last year before therapy initiation are shown in shades of blue, with times between the first and last year shown as orange, yellow, and green. (TIF) [file ppat.1011974.s020.tif]

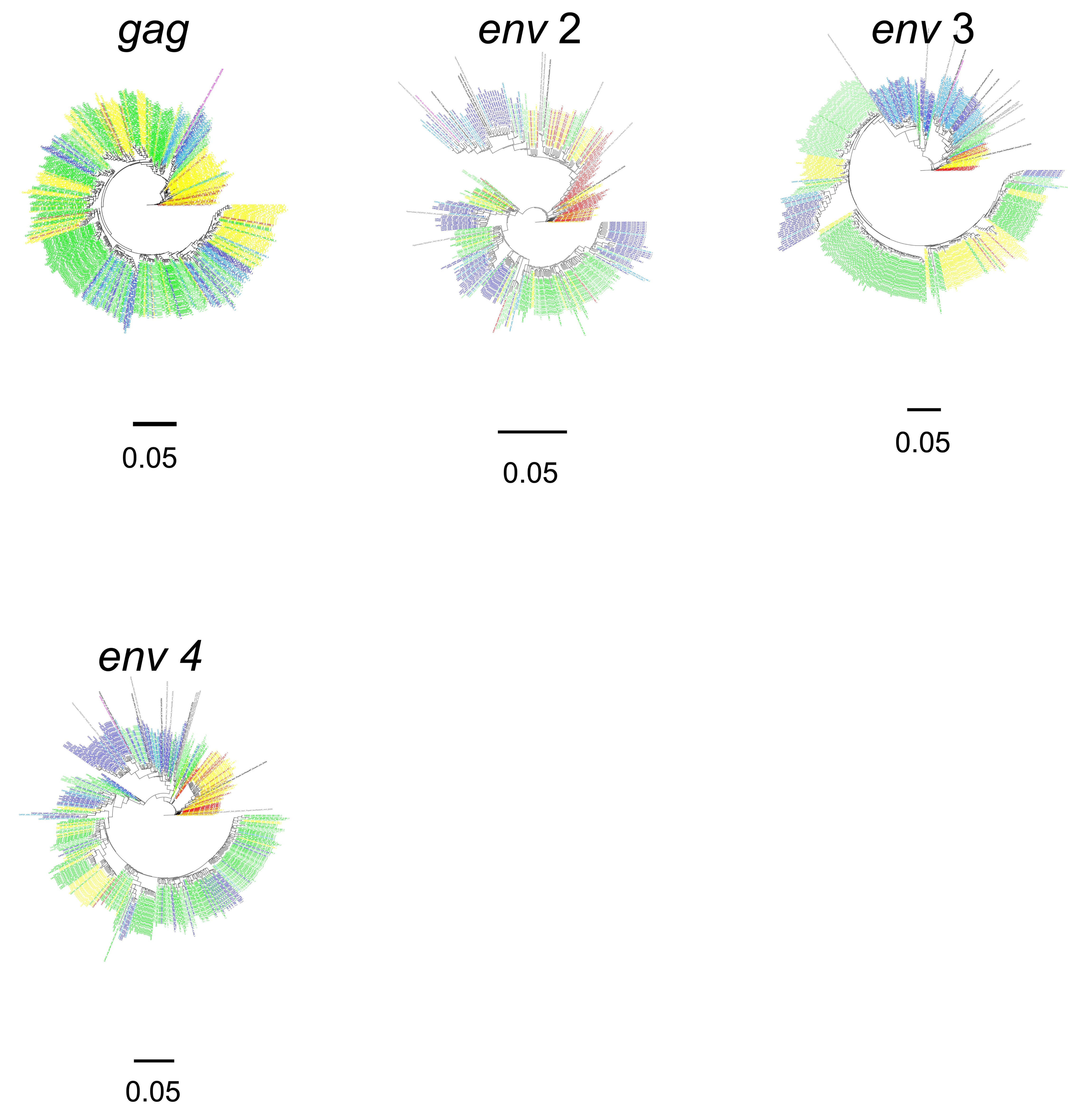

Supplement: S18 Fig — Approximately Maximum-Likelihood trees were used for each of the gene regions. OGV sequences are shown in magenta and proviral sequences are shown in black (non-hypermutated viral DNA) and gray (hypermutated viral DNA). Sequences generated from plasma collected within the first year of diagnosis are shown in shades of red, within the last year before therapy initiation are shown in shades of blue, with times between the first and last year shown as orange, yellow, and green. (TIF) [file ppat.1011974.s021.tif]

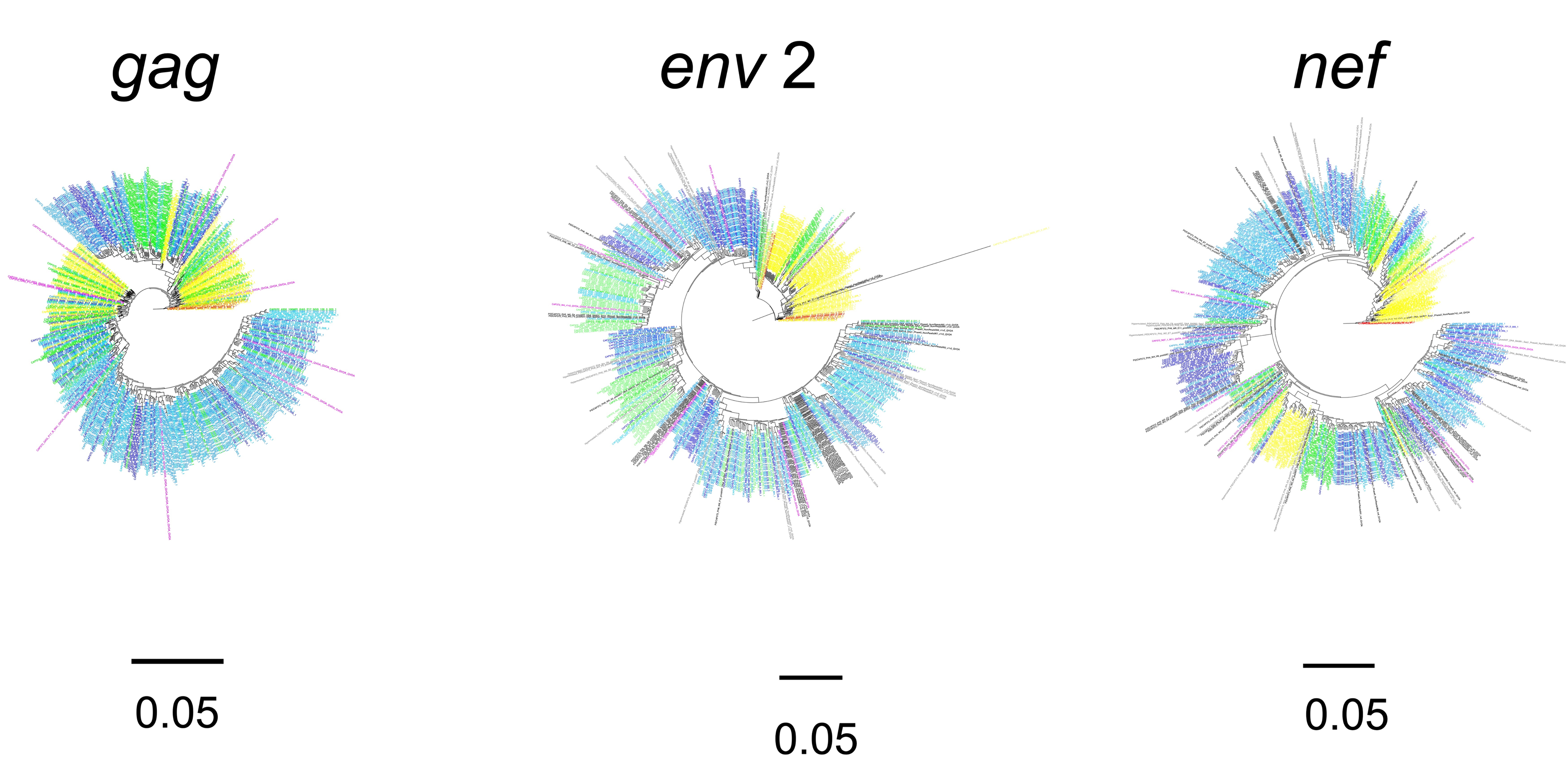

Supplement: S19 Fig — Approximately Maximum-Likelihood trees were used for each of the gene regions. OGV sequences are shown in magenta and proviral sequences are shown in black (non-hypermutated viral DNA) and gray (hypermutated viral DNA). Sequences generated from plasma collected within the first year of diagnosis are shown in shades of red, within the last year before therapy initiation are shown in shades of blue, with times between the first and last year shown as orange, yellow, and green. (TIF) [file ppat.1011974.s022.tif]

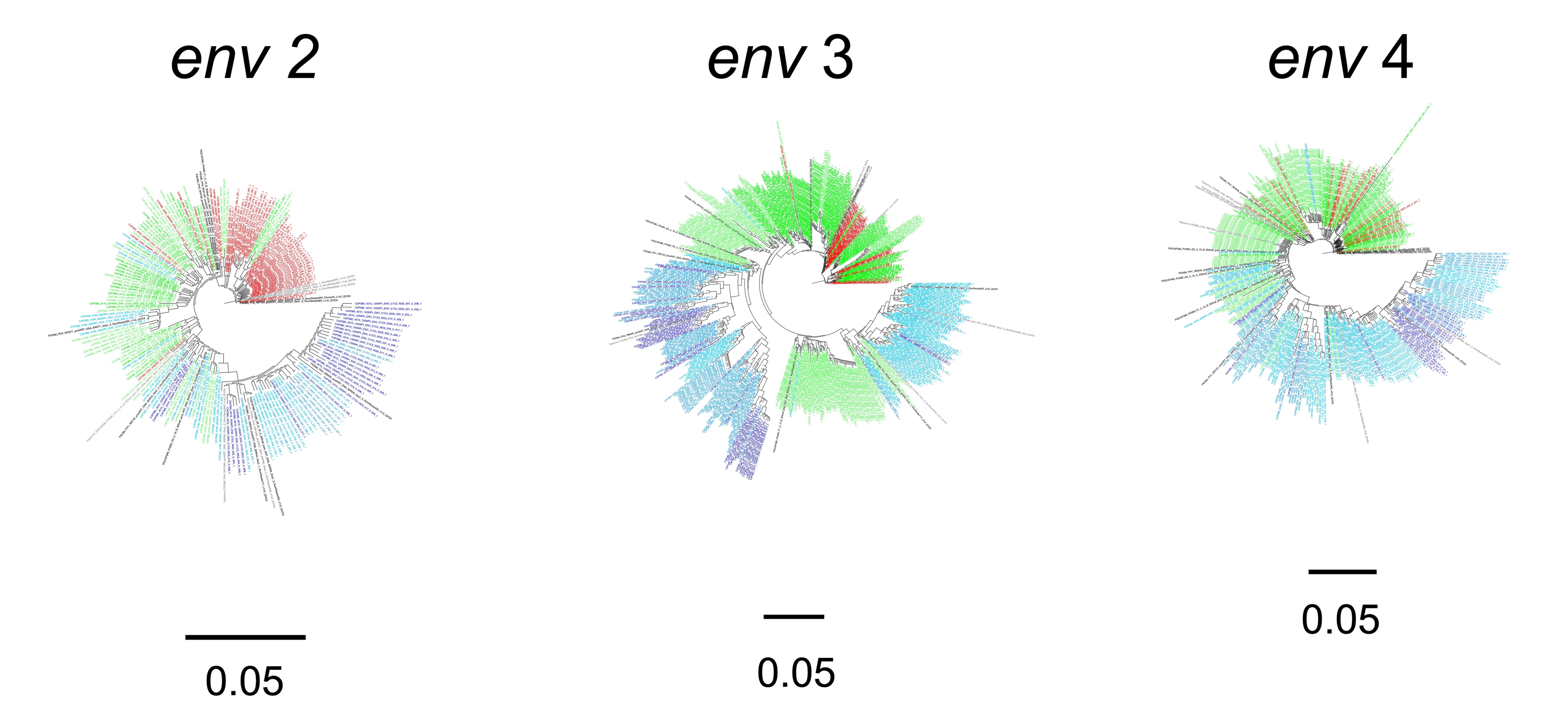

Supplement: S20 Fig — Approximately Maximum-Likelihood trees were used for each of the gene regions. OGV sequences are shown in magenta and proviral sequences are shown in black (non-hypermutated viral DNA) and gray (hypermutated viral DNA). Sequences generated from plasma collected within the first year of diagnosis are shown in shades of red, within the last year before therapy initiation are shown in shades of blue, with times between the first and last year shown as orange, yellow, and green. (TIF) [file ppat.1011974.s023.tif]

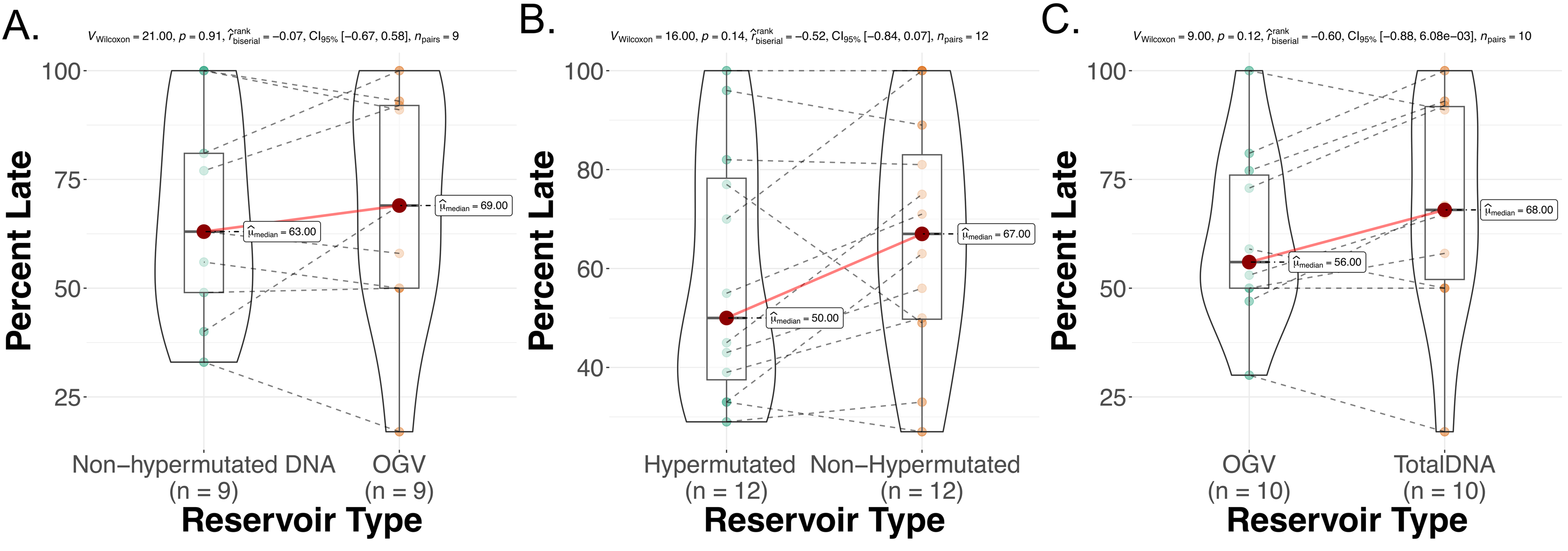

Supplement: S21 Fig — Comparisons of the percent of reservoir sequences that entered the reservoir during the year before the initiation of therapy (i.e. the percent late virus) did not detect differences in when different types of variants entered the long-lived reservoir. A. Non-hypermutated vDNA and OGVs (Wilcoxon Matched-Pairs Rank Sum; P = 0.91). B. Hypermutated vDNA (masked) and non-hypermutated vDNA (Wilcoxon Matched-Pairs Rank Sum; P = 0.14). C. OGVs and total vDNA (Wilcoxon Matched-Pairs Rank Sum; P = 0.12). Each line represents one participant and analyses are restricted to participants with at least six sequences of the types being compared. (TIF) [file ppat.1011974.s024.tif]
