# Supplementary material for: The timing of HIV-1 infection of cells that persist on therapy is not strongly influenced by replication competency or cellular tropism of the provirus
Source: PLoS Pathog. 2024 Feb 29;20(2):e1011974. doi: 10.1371/journal.ppat.1011974 (PMC10931466; doi:10.1371/journal.ppat.1011974)
Supplement: S22 Fig — In order to determine whether masking reduces the dataset too much to accurately date reservoir sequences we examined whether masking APOBEC3G/F recognition sequences precludes accurate dating of control sequences that are not hypermutated (OGVs). Masking did not significantly alter the estimation of the percent of control sequences that form late (Wilcoxon Matched-Pairs Rank Sum; P = 0.68). (DOCX) [file ppat.1011974.s025.docx]

**
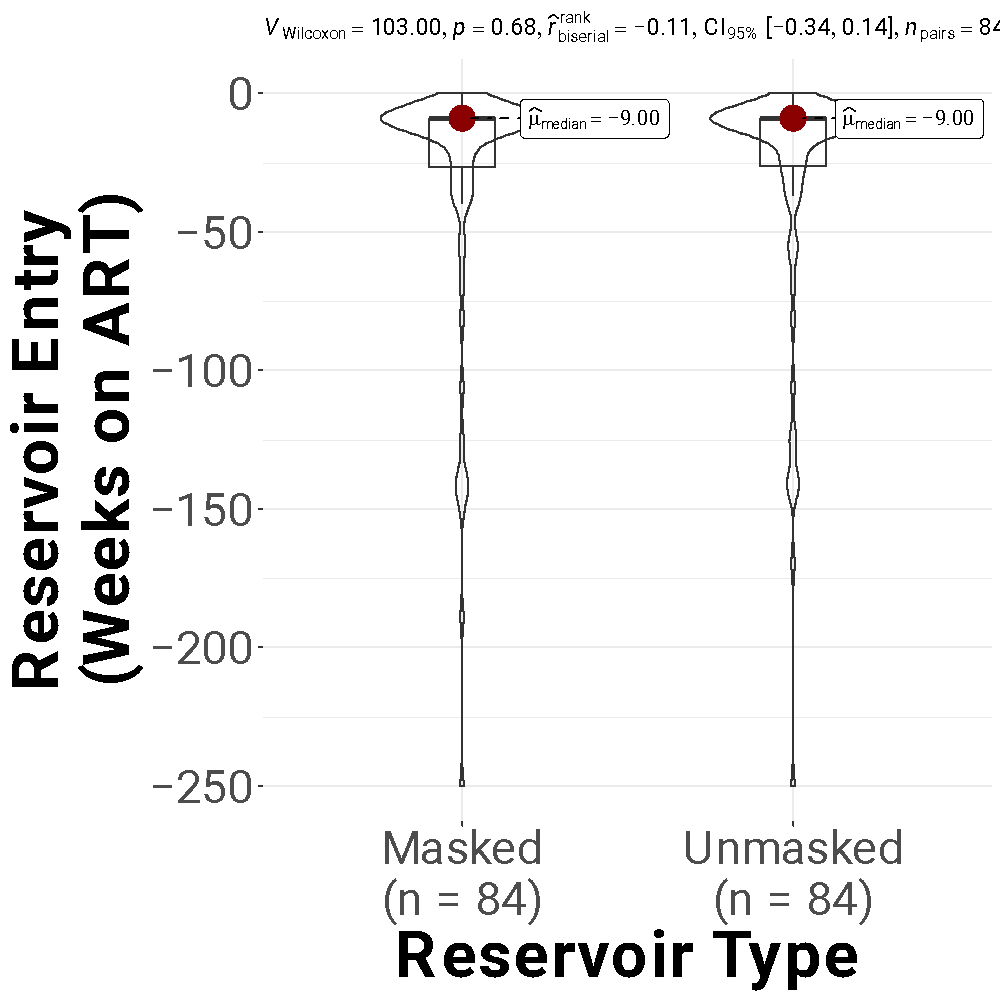
**

**S22 Fig. Masking Potentially Hypermutated Positions Does Not Preclude Precise Estimation of When Variants Enter the Long-lived Reservoir.** In order to determine whether masking reduces the dataset too much to accurately date reservoir sequences we examined whether masking APOBEC3G/F recognition sequences precludes accurate dating of control sequences that are not hypermutated (OGVs). Masking did not significantly alter the estimation of the percent of control sequences that form late (Wilcoxon Matched-Pairs Rank Sum; P=0.68).
